# Expanding Characterized Diversity and the Pool of Complete Genome Sequences of *Methylococcus* Species, the Bacteria of High Environmental and Biotechnological Relevance

**DOI:** 10.3389/fmicb.2021.756830

**Published:** 2021-10-06

**Authors:** Igor Y. Oshkin, Olga V. Danilova, Sergey Y. But, Kirill K. Miroshnikov, Ruslan Z. Suleimanov, Svetlana E. Belova, Ekaterina N. Tikhonova, Nikolai N. Kuznetsov, Valentina N. Khmelenina, Nikolai V. Pimenov, Svetlana N. Dedysh

**Affiliations:** ^1^Winogradsky Institute of Microbiology, Research Center of Biotechnology, Russian Academy of Sciences, Moscow, Russia; ^2^G. K. Skryabin Institute of Biochemistry and Physiology of Microorganisms, Pushchino Scientific Center for Biological Research, Russian Academy of Sciences, Pushchino, Russia

**Keywords:** *Methylococcus*, methanotrophic bacteria, cultivation studies, comparative genomics, pan-genome analysis, growth on methane, phage-associated regions

## Abstract

The bacterial genus *Methylococcus*, which comprises aerobic thermotolerant methanotrophic cocci, was described half-a-century ago. Over the years, a member of this genus, *Methylococcus capsulatus* Bath, has become a major model organism to study genomic and metabolic basis of obligate methanotrophy. High biotechnological potential of fast-growing *Methylococcus* species, mainly as a promising source of feed protein, has also been recognized. Despite this big research attention, the currently cultured *Methylococcus* diversity is represented by members of the two species, *M. capsulatus* and *M. geothermalis*, while finished genome sequences are available only for two strains of these methanotrophs. This study extends the pool of phenotypically characterized *Methylococcus* strains with good-quality genome sequences by contributing four novel isolates of these bacteria from activated sludge, landfill cover soil, and freshwater sediments. The determined genome sizes of novel isolates varied between 3.2 and 4.0Mb. As revealed by the phylogenomic analysis, strains IO1, BH, and KN2 affiliate with *M. capsulatus*, while strain Mc7 may potentially represent a novel species. Highest temperature optima (45–50°C) and highest growth rates in bioreactor cultures (up to 0.3h^−1^) were recorded for strains obtained from activated sludge. The comparative analysis of all complete genomes of *Methylococcus* species revealed 4,485 gene clusters. Of these, pan-genome core comprised 2,331 genes (on average 51.9% of each genome), with the accessory genome containing 846 and 1,308 genes in the shell and the cloud, respectively. Independently of the isolation source, all strains of *M. capsulatus* displayed surprisingly high genome synteny and a striking similarity in gene content. Strain Mc7 from a landfill cover soil differed from other isolates by the high content of mobile genetic elements in the genome and a number of genome-encoded features missing in *M. capsulatus*, such as sucrose biosynthesis and the ability to scavenge phosphorus and sulfur from the environment.

## Introduction

The genus *Methylococcus* was described in 1966 by Foster and Davis, who isolated a culture of coccus-shaped bacteria capable of growth in a mineral medium with methane (CH_4_) as the only carbon source from a sludge sample of the Austin municipal sewage plant, Texas (Foster and Davis, 1966). After description of “*Pseudomonas methanica*” (Dworkin and Foster, 1958; currently known as *Methylomonas methanica*), this was the second documented case of an organism, which consumes CH_4_ for growth. The original description of the genus *Methylococcus* characterized its members as non-motile, obligately aerobic, Gram-negative cocci, approximately 1μm in diameter, with a characteristic diplococcoid arrangement, which utilize methane or methanol as the only carbon sources. The species epithet *capsulatus* was assigned to these bacteria in order to denote the presence of capsules revealed by staining cell specimens with the India ink. The originally described isolate, strain Texas, was defined as the type strain of the species *Methylococcus capsulatus*. Several later proposed species of this genus, such as “*M. bovis*,” “*M. chroococcus*,” “*M. luteus*,” “*M. vinelandii*,” and “*M. whittenburyi*” (Romanovskaya et al., 1981), were transferred to the genus *Methylobacter* after detailed re-examination of their characteristics (Bowman et al., 1993). The species *M. thermophilus* (Malashenko et al., 1975) has been retained in the genus *Methylococcus* (Bowman et al., 1993), but the type strain of this species is no longer available from culture collections. The single strain making up the species *Methylococcus mobilis* (Hazeu et al., 1980) has also been lost. Apparently, all *Methylococcus* species are difficult to preserve (Bowman, 2015), which is the main reason behind poor availability of these bacteria from both public and laboratory culture collections. Recently described *Methylococcus geothermalis* (Awala et al., 2020) is the second described species of the genus, which is currently available in culture besides *M. capsulatus*.

Members of the genus *Methylococcus* are thermotolerant or moderately thermophilic bacteria, with optimal growth between 40–60°C (Bowman, 2015). They have been isolated from sewage sludge, sediments of rivers and ponds, wastewater of coal mines, and geothermal fields (Malashenko et al., 1975; Bowman et al., 1993). As suggested by culture-independent studies, *Methylococcus*-like methanotrophs can also be detected in landfill cover soils (Gebert et al., 2009), geothermal soils (Gagliano et al., 2014), and hot springs (Kizilova et al., 2014; Houghton and Stewart, 2020). Cells of these bacteria possess both known types of methane monooxygenase (MMO), which catalyzes the oxidation of methane to methanol, i.e., particulate (pMMO) and soluble (sMMO) forms of this enzyme. Taxonomically, the genus *Methylococcus* belongs to the gammaproteobacterial family *Methylococcaceae*, which accommodates the so-called type I methanotrophs. The latter utilize the ribulose monophosphate pathway (RuMP) for formaldehyde assimilation, while alphaproteobacterial type II methanotrophs employ the serine pathway. One specific feature of the metabolic organization of *Methylococcus* and *Methylococcus*-related methanotrophs is that, in addition to RuMP pathway, they also possess the Calvin–Benson–Bassham (CBB) cycle. This was one of the key reasons to consider these bacteria as representing a separate, type X, methanotrophs (Whittenbury, 1981; Whittenbury and Dalton, 1981). Most cultured representatives of the genus *Methylococcus* did not receive much research attention. One clear exception is *M. capsulatus* strain Bath, which was extensively studied and gradually became probably the best characterized of the aerobic methanotrophs in terms of genetics and physiology (Anthony, 1983; Dalton, 2005; Murrell, 2010). All major insights into MMO structure and function were made by using *M. capsulatus* Bath. Those include studies on resolving the structure of soluble and particulate MMO (Colby and Dalton, 1978; Rosenzweig et al., 1993; Zahn and DiSpirito, 1996; Müller et al., 2002; Chatwood et al., 2004; DiSpirito et al., 2004; Lieberman and Rosenzweig, 2005; Balasubramanian et al., 2010; Ross et al., 2019) as well as control of the MMOs expression based on copper-to-biomass ratios known as a “copper switch” (Stanley et al., 1983; Kao et al., 2004; Larsen and Karlsen, 2016). *Methylococcus capulatus* Bath was also the first methanotrophic organism for which a genome was published (Ward et al., 2004). This bacterium has a relatively small genome (3.3Mb) compared to other methanotrophs. The genome contains two copies of the gene clusters encoding pMMO and one copy of the sMMO gene cluster as well as *mxa* and *xox* operons encoding two alternative types of methanol dehydrogenases (MDHs). Primary route for carbon assimilation is the RuMP pathway although genes for the serine pathway, and the CBB cycle are also present. Routes for nitrogen assimilation include ammonia assimilation enzymes (glutamine synthetase, glutamate synthase, and alanine dehydrogenase) and nitrogenase. Genome contains genes encoding all tricarboxylic acid (TCA) cycle enzymes, but the absence of 2-oxoglutarate dehydrogenase activity *in vitro* still suggests that the Krebs cycle cannot operate in *M. capsulatus* Bath (Murrell, 2010). To date, two metabolic models were published based on the genome sequence of *M. capsulatus* Bath (Gupta et al., 2018; Lieven et al., 2018). Experimental verification of metabolic features of this methanotroph was performed in transcriptomic and proteomic studies (Kao et al., 2004; Larsen and Karlsen, 2016).

High biotechnological potential of fast-growing *Methylococcus* species has also been recognized. In particular, the conversion of methane to biomass by *M. capsulatus* has been exploited for large-scale commercial production of microbial proteins by fermentation (Skrede et al., 1998). The biomass of *M. capsulatus* Bath was approved as a promising source of protein based on criteria such as amino acid composition, digestibility, and animal performance and health (Skrede et al., 2009). Single-cell protein based on *M. capsulatus* is suitable for fish feeding as besides good nutritional value it was proved to prevent soybean meal-induced enteritis in Atlantic salmon (Romarheim et al., 2011). *Methylococcus capsulatus* Bath was also explored for the potential of generating electricity directly from methane (Jawaharraj et al., 2021).

Despite the big research interest in methanotrophs of the genus *Methylococcus*, the number of currently available good-quality genome assemblies of *Methylococcus* species is limited to those of *M. capulatus* Bath and *M. geothermalis* IM1^T^. Besides these two finished genomes, 24 draft genome assemblies for members of the genus *Methylococcus* have been deposited in GenBank. Of these, 21 assemblies represent metagenome-assembled genomes (MAGs). This lack of good-quality genome sequences limits the potential of comparative genomic studies as well as our understanding of the variability of genome-encoded features within the genus *Methylococcus*.

This study was initiated in order to expand the narrow range of currently available *Methylococcus* cultures by isolating novel strains of these bacteria from various habitats. Our work resulted in obtaining four new strains of the genus *Methylococcus* and the corresponding complete genomes. These newly obtained genomes as well as all available complete genome sequences of other *Methylococcus* strains were compared in order to examine variability of genome-encoded features within this genus. Despite being isolated from geographically remote habitats, all strains of the species *M. capsulatus* displayed surprisingly high genome synteny and a striking similarity in gene content. By contrast, one novel isolate from a landfill cover soil possessed a number of genome-encoded features missing in *M. capsulatus*, such as sucrose biosynthesis and the ability to scavenge phosphorus and sulfur from the environment. This isolate may potentially represent a novel species of the genus *Methylococcus*.

## Materials and Methods

### Isolation Procedures

In this study, activated sludge, freshwater sediment, and landfill cover soil were used as sources for isolation of new *Methylococcus* strains. Activated sludge samples were collected in bottles (500ml each) from two municipal wastewater treatment plants in Moscow, Russia. Freshwater sediment samples were collected in 50ml falcon tubes from beneath shallow water in an unnamed lake in Krasnodar region, Russia. Surface (depth of 0–5cm) soil samples were collected from a landfill site in Khanty-Mansiysk region. Aliquots of collected samples were used as inoculum to obtain enrichment cultures of methanotrophic bacteria. The latter were obtained using modified AMS medium (mAMS), containing (in grams per liter of distilled water) NH_4_Cl, 0.1; MgSO_4_×7H_2_O, 0.2; CaCl_2_×2H_2_O, 0.02; 100mM phosphate buffer, pH 5.8, 1% (vol/vol); and trace element solution 0.1% (vol/vol), containing the following (g/L): EDTA, (in grams per liter) EDTA, 5; FeSO_4_×7H_2_O, 2; ZnSO_4_×7H_2_O, 0.1; MnCl_2_×4H_2_O, 0.03; CoCl_2_×6H_2_O, 0.2; CuSO_4_×5H_2_O, 0.1; NiCl_2_×6H_2_O, 0.02; and Na_2_MoO_4_, 0.03. After inoculation, 500ml bottles were sealed with silicone rubber septa, and methane was added aseptically using a syringe equipped with a disposable filter (0.22μm) to achieve a 10–20% mixing ratio in the headspace. Bottles were incubated on a rotary shaker (150 r.p.m.) at 42°C. After 1week of incubation, the cultures enriched with methanotrophic bacteria were subjected to serial dilutions. After several serial dilution steps, cell suspensions were plated on agar-solidified mAMS medium. The plates were incubated at 42°C in desiccators containing approximately 30% methane in air. The colonies appearing on the plates were picked and restreaked on the same agar medium. The set of finally selected colonies was subjected to several additional serial dilution steps in a liquid mAMS medium at 42°C until isolates of methanotrophic bacteria were obtained. Culture purity was verified by examination using phase-contrast microscopy and by plating on 10-fold diluted Luria–Bertani agar (1.0% tryptone, 0.5% yeast extract, 1.0% NaCl).

### Morphological Characterization and Growth Tests

Prior to cell size measurements and growth experiments, the isolates were maintained in liquid mAMS medium with 20% (*v*/*v*) CH_4_ at 42°C for 10days, with regular transfers each 2–3days. *Methylococcus capulatus* Bath was used as a reference organism in all experiments. Morphological observations and cell-size measurements were made with a Zeiss Axioplan 2 microscope and Axiovision 4.2 software (Zeiss). Cell sizes were measured for 20 randomly selected cells of all strains. Comparative analysis of growth characteristics of the isolates was performed by monitoring their growth dynamics in liquid mineral medium mAMS with 20% methane in the headspace within the temperature range of 25–55°C. Variations in the pH were achieved by mixing 0.1 M solutions of H_3_PO_4_, KH_2_PO_4_, and K_2_HPO_4_. All incubations were performed in triplicate. Growth dynamics was determined by measuring OD_600_ of the cultures on an Eppendorf Biophotometer AG spectrophotometer. The ability to grow on methanol was tested in mAMS medium containing 0.01–3% (*v*/*v*) CH_3_OH. Nitrogen sources were tested by replacing NH_4_Cl in mAMS with 0.01% (*w*/*v*) NaNO_3_, NaNO_2_, urea, methylamine, glutamine, glycine, alanine, peptone, and yeast extract. Growth was examined after 3days of incubation. Salt tolerance was examined by adding NaCl to mAMS medium in concentrations of 0–2% (*w*/*v*).

Experiments on continuous cultivation were conducted in a 1.5L bioreactor filled with 1L of mineral medium. The inlet gases were methane with a flow rate of 100mlmin^−1^ and air pumped in with an air compressor with a flow rate of 500mlmin^−1^. The pH level of 5.6 was controlled by titration with 0.8% NH_4_OH solution. Agitation was kept constant at 1,000 r.p.m. All gases were filtered with a 0.22μm sterile membrane. Each of the growth experiments started as a batch cultivation. When the culture reached exponential phase, bioreactor was switched to a continuous mode at a dilution rate of 0.05h^−1^. The later was increased with an increment of 0.05h^−1^ every 1–2days until a culture washout was observed. After that, the dilution rate was decreased by 0.05h^−1^ and bioreactor was operated in a continuous mode for the next 5days.

### Hydrogen Utilization

Cells of *Methylococcus* strains were pre-grown to the exponential phase in 120-ml serum bottles containing 20ml of mAMS medium. Hydrogen utilization was tested with and without the addition of methane under fully aerobic conditions. Headspace of experimental bottles was filled with either 2% CO_2_, 2% H_2_ and 20% CH_4_ or only 2% H_2_ and 2% CO_2_. Strains were cultivated for 24h using the same cultivation conditions as described above. Control treatments contained 2% CO_2_ or 20% CH_4_ and 2% CO_2_ in the headspace. Methane and hydrogen were measured by gas chromatography at the beginning of the experiment and after 24h of cultivation. The optical density was measured at 600nm wave length (OD_600_) using Eppendorf Biophotometer UV/Vis spectrophotometer (Eppendorf, Germany).

### DNA Extraction

Cultures of new isolates were grown in the liquid mAMS as described above. The cells were harvested after incubation at 42°C on a rotary shaker at 150rpm for 2days. Genomic DNA extraction was done using the standard CTAB and phenol-chloroform protocol (Wilson, 2001).

### Genome Sequencing and Annotation

Genomic libraries suitable for MiSeq sequencing were prepared with a NEBNext ultra II DNA Library kit (New England Biolabs). On average, a total of 1.68 million paired-end reads (2×300; 250nt) were obtained for each genome. Nanopore sequencing library was prepared using the 1D ligation sequencing kit (SQK-LSK108, Oxford Nanopore, United Kingdom). Sequencing was performed on an R9.4 flow cell (FLO-MIN106) using MinION device. Hybrid assembly of short and long reads was performed using Unicycler v.0.4.8 (Wick et al., 2017). Assemblies were evaluated with Quast 5.0 (Gurevich et al., 2013) and Busco 5.1.2 (Simão et al., 2015).

Annotation was performed using the RAST server (Aziz et al., 2008) and Prokka (Seemann, 2014). In addition, the presence of genes encoding enzymes of the primary and central metabolism as well as some secondary metabolic pathways discussed below was verified manually by web version of NCBI blastp using 35% identity and 50% coverage of amino acid sequence as a cut-off. The same criteria were used for searching genes of interest in the genomes of other methylotrophs. Assembled genomes have been deposited in NCBI GenBank under the accession numbers CP079095–CP079098.

### Phylogenomic Analysis

A genome-based tree for members of the *Methylococcus* group was reconstructed using the Genome Taxonomy Database and GTDB-toolkit,^1^ release 04-RS89. The maximum likelihood phylogenetic tree was constructed using MegaX software (Kumar et al., 2018). The Pyani program was used to estimate average nucleotide identities across *Methylococcus* genomes (Pritchard et al., 2016). The resulting distance matrices were further visualized as a heatmap in R (R Core Team, 2020).

### Pan-Genome Analysis

The pan-genome was reconstructed using microbial pangenomics workflow in Anvi’o (Eren et al., 2015). The genomes were annotated in Prokka, after which the genes were organized using MCL algorithm into core, shell, and singleton clusters (Distance: Euclidean; Linkage: Ward). The core, shell, and singleton genes were separately annotated by BLASTp against the NCBI COG database using eggNOG-mapper (Cantalapiedra et al., 2021). Heatmap based on the annotated COG functions of the core and singleton gene clusters was then plotted in R. The Tettelin best-fit curves of the core- and pan-genomes were constructed using OMCL v1.4 implemented in GET_HOMOLOGUES pipeline (Contreras-Moreira and Vinuesa, 2013).

Whole-genome synteny was computed using Sibelia (Minkin et al., 2013). Synteny blocks were visualized using Circos (Krzywinski et al., 2009).

### Identification of the Mobile Genetic Elements and Prophage Regions

Insertion sequences (IS) were identified and classified into IS families using ISsaga pipeline (Varani et al., 2011) with IS finder database (Siguier et al., 2006) and ISEScaner pipeline (Xie and Tang, 2017). ICEfinder and ICEBerg v2.0 were used for IME detection (Liu et al., 2018b). Integron Finder (Cury et al., 2016) was used to identify regions containing integrons. Potential prophage regions were searched with PHASTER server^2^ using the settings described in Arndt et al. (2016). Gene functions were determined by homology with known viral proteins in the NCBI GenBank database and the VirFam package^3^ using the settings described in Lopes et al. (2014).

## Results and Discussion

### Identification and Characterization of New *Methylococcus* Strains

In total, four novel isolates of *Methylococcus*-like methanotrophs were obtained and characterized in this study. Strains IO1 and KN2 were isolated from activated sludge samples of the Moscow municipal sewage plant (Table 1). These isolates displayed nearly identical 16S rRNA gene sequences and were closely related to *M. capsulatus* Bath (99.93–99.97% 16S rRNA gene sequence similarity). Strain BH was obtained from a sediment of an unnamed freshwater lake in Krasnodar region, South Russia and was also closely related to *M. capsulatus* Bath (99.02% 16S rRNA gene sequence similarity). Finally, strain Mc7 was isolated from a landfill cover soil in Khanty-Mansiysk region, West Siberia, Russia. In contrast to other three isolates, strain Mc7 displayed highest 16S rRNA gene sequence similarity (98.56%) to *M. geothermalis* IM1^T^.

**Table 1:** Isolation sources and some characteristics of new *Methylococcus* isolates.

Strain	Isolation source	Cell size, μm	Growth temperature range (optimum), °C	Growth on methanol, % CH_3_OH	NaCl tolerance, %	pH growth range (optimum)	Growth rate in batch culture/bioreactor, h^−1^
IO1	Activated sludge, Moscow, Russia	1.14±0.02	28–52 (48)	0.05–0.1 (trace)	0.75	5.5–7.5 (6.5–7.0)	0.19/0.26
KN2	Activated sludge, Moscow, Russia	1.12±0.03	25–53 (48–50)	0.05–3.0	0.75	4.6–8.5 (6.5–8.0)	0.22/0.30
BH	Lake sediment, Krasnodar region, Russia	1.12±0.02	25–52 (42)	0.05–1.5	0.75	5.5–8.0 (6.5–7.2)	0.18/0.28
Mc7	Landfill cover soil, Khanty-Mansiysk, Russia	1.59±0.03	28–53 (40)	0.05–0.1 (trace)	1.0	5.5–7.5 (6.5–7.0)	0.23/0.27

**Figure 1:**
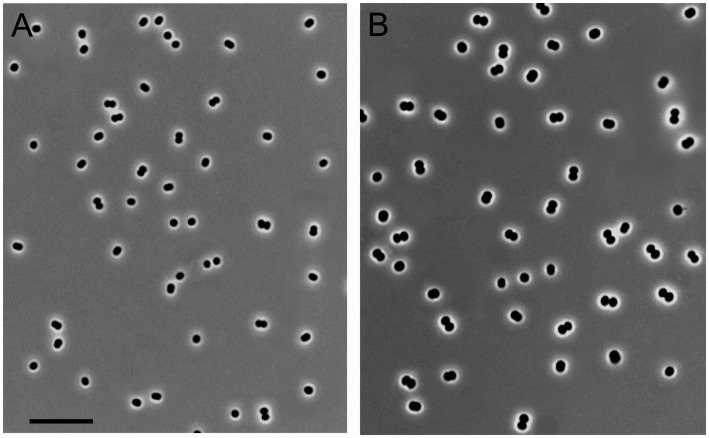
Cell morphology of strains KN2 **(A)** and Mc7 **(B)** grown for 1day in liquid medium mAMS with methane. Marker, 10μm.

Four novel isolates were represented by non-motile, Gram-negative cocci, with a characteristic diplococcoid arrangement (Figure 1). Cell sizes of strains IO1, KN2, and BH were the same as those reported for *M. capsulatus*, approximately 1μm in diameter (Table 1; Figure 1A). Cell sizes of strain Mc7 (1.6μm in diameter; Figure 1B) were larger than those of other three strains. The colonies formed by these isolates on agar mineral medium after 2weeks of incubation with 20% (*v*/*v*) methane were small (1–2mm in diameter), round, milk-white (for strain Mc7) or cream colored (for strains IO1, KN2, and BH).

Methane and methanol were used as growth substrates, although the isolates differed in their ability to grow on methanol. Similar to *M. capsulatus* Bath, strains IO1 and Mc7 displayed only a trace growth (up to OD_600_ 0.10–0.12) in a relatively narrow range of CH_3_OH concentrations (Table 1). By contrast, strains KN2 and BH were capable of a relatively good growth (up to OD_600_ 0.3) on methanol in a wide range of concentrations. The isolates were quite uniform with regard to their pH preferences (Table 1). Most strains grew in the pH range of 5.5–8.0. Strain KN2 demonstrated slightly better adaptation to moderately acidic conditions growing between pH 4.6 and 8.5. All strains used nitrate and ammonium as nitrogen sources. In addition, strains BH and KN2 were able to utilize glutamine, while strain IO1 showed weak growth on peptone as a nitrogen source. Strain Mc7 displayed highest tolerance to NaCl, up to 1% (*w*/*v*), while other isolates tolerated up to 0.75% (*w*/*v*) NaCl. For comparison, NaCl tolerance determined in our experiments for *M. capsulatus* Bath was 0.5% (*w*/*v*).

The ability to utilize hydrogen was tested using incubations with either H_2_ and CO_2_ or H_2_, CO_2_ and CH_4_ in the headspace (see Materials and Methods). No growth as well as no H_2_ consumption were observed in the absence of CH_4_, with H_2_ as the only energy source. With the only exception of strain KN2, the presence of H_2_ in addition to CH_4_ increased the growth yield of all strains examined in this study by 2–20% (Supplementary Figure S1A) as well as the corresponding CH_4_ consumption (Supplementary Figure S1B). H_2_ utilization in the presence of CH_4_ was observed for all studied strains, including strain KN2 (Supplementary Figure S1C). These results agree well with the previous report of the absence of autotrophic growth of *M. capsulatus* Bath in liquid medium with H_2_ and CO_2_ (Baxter et al., 2002; Henard et al., 2021). Apparently, *Methylococcus* strains are capable of using H_2_ as an alternative energy source during their growth on methane. Recently, the ability to grow mixotrophically on H_2_ and CH_4_ was also reported for verrucomicrobial (*Methylacidiphilum* sp. RTK17.1) and proteobacterial (*Methylocystis* sp. strain SC2) methanotrophs (Carere et al., 2017; Mohammadi et al., 2019; Hakobyan et al., 2020).

**Figure 2:**
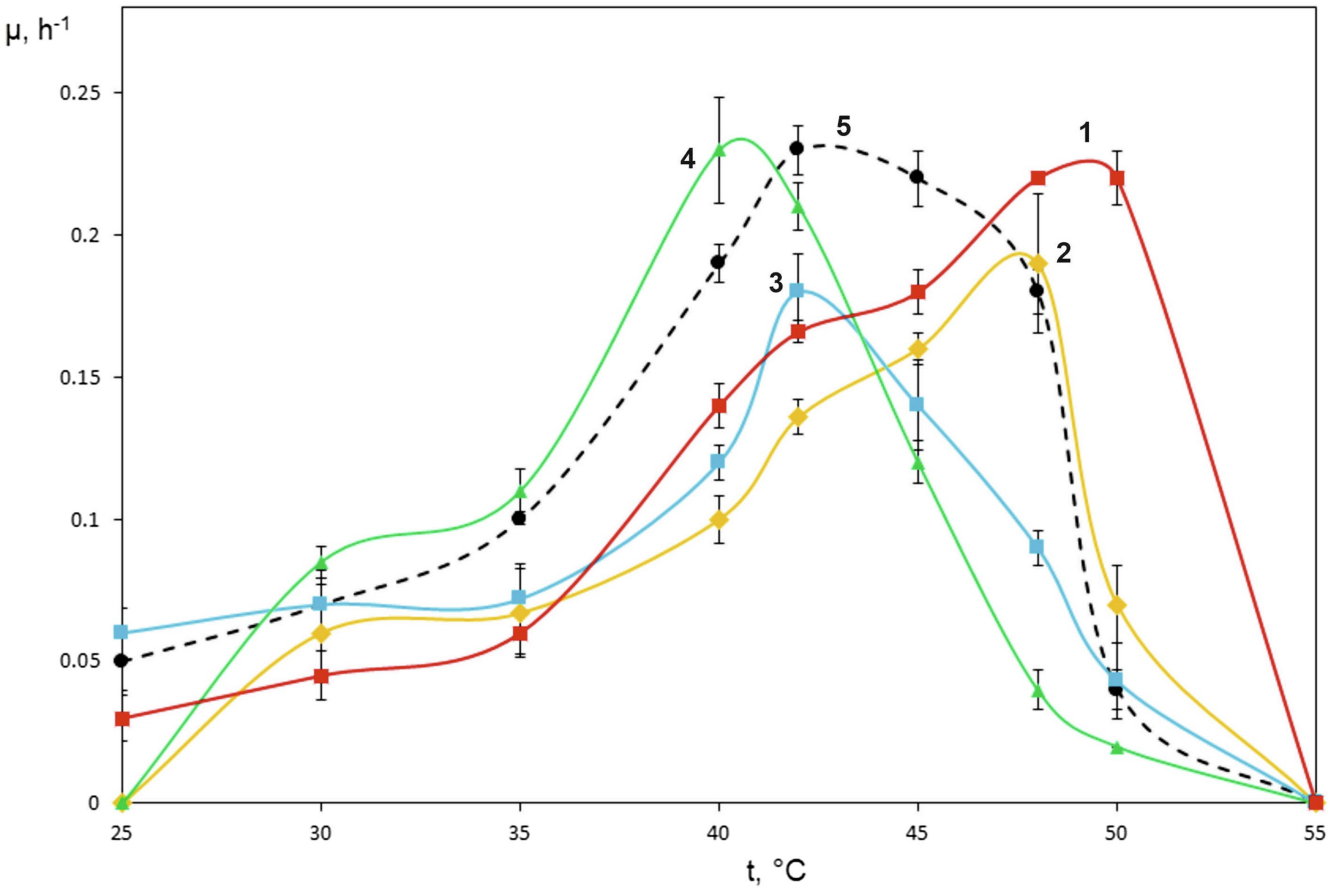
Specific growth rates of novel isolates on methane as dependent on incubation temperature. *Methylococcus capsulatus* Bath was used as a reference organism. 1, strain KN2, 2, strain IO1, 3, BH, 4, Mc7, and 5, *Methylococcus capsulatus* Bath.

The special attention in our study was given to assessing growth characteristics of novel *Methylococcus* isolates. The results of measuring specific growth rates of novel isolates and *M. capsulatus* Bath within the temperature range of 25–55°C are shown in Figure 2. The two strains obtained from sludge samples, IO1 and KN2, were clearly more thermotolerant than *M. capsulatus* Bath and displayed growth optima at 48–52°C. The lowest temperature growth optimum of 40°C was revealed for strain Mc7, which is reasonable given that it was isolated from a landfill soil in West Siberia. Highest specific growth rates, 0.22–0.23h^−1^, which were comparable to that of *M. capsulatus* Bath, were recorded for strains KN2 and Mc7. We also performed additional experiments in order to examine specific growth rates of novel isolates during continuous cultivation on methane in 1.5L bioreactor. The highest specific growth rate in a fermenter culture (0.3h^−1^) was recorded for strain KN2. Other strains also demonstrated stable growth under conditions of continuous cultivation. Their growth rates, however, were below that demonstrated by strain KN2 (Table 1).

### Genome Sequencing and Assembly

The genomes of four novel isolates were sequenced using a hybrid approach. Oxford Nanopore sequencing yielded 192,565–278,437 reads with a total length of 1.05–1.59 Gb (Supplementary Table S1). Sequencing on Illumina MiSeq platform generated a total of 415,244–3,477,384 paired-end reads, with a mean read length of 250bp. Both short and long reads were combined to perform a hybrid assembly using Unicycler, resulting in circular genomes. Genome characteristics of new isolates are summarized in Table 2.

**Table 2:** General genome features of new isolates and publicly available complete *Methylococcus* genomes.

	Strain KN2	Strain BH	Strain Mc7	Strain IO1	*Methylococcus capsulatus* Bath	*Methylococcus geothermalis* IM1^T^
Genome size (Mb)	3.6	3.2	4.0	3.3	3.3	3.4
Contigs	1	1	1	1	1	1
G+C content (mol %)	63.49	63.52	63.44	63.49	63.60	63.25
CDS	3,289	2,912	3,752	3,008	3,043	3,091
Repeat region	2	2	6	2	2	3
tRNA	48	50	48	50	49	48
5S, 16S, 23S	2, 2, 2	2, 2, 2	2, 2, 2	2, 2, 2	2, 2, 2	2, 2, 2
pMMO operon	2	2	2	2	2	2
sMMO operon	1	1	1	1	1	1
Complete IS elements	17	23	78	26	33	45

Genome sizes varied from 3.2Mb in strain BH to 4.0Mb in strain Mc7. The DNA G+C content was highly similar in all examined genomes and constituted 63.44–63.52%. Each genome contained two copies of rRNA operon, two copies of pMMO, and one copy of sMMO. The number of protein-coding genes varied between 2,912 and 3,752. No plasmids were detected. Lowest number (17) of insertion (IS) elements was detected in strain KN2, while substantially higher number of IS elements (78) was observed in strain Mc7.

### Genome-Based Phylogeny and Genome-to-Genome Comparison

**Figure 3:**
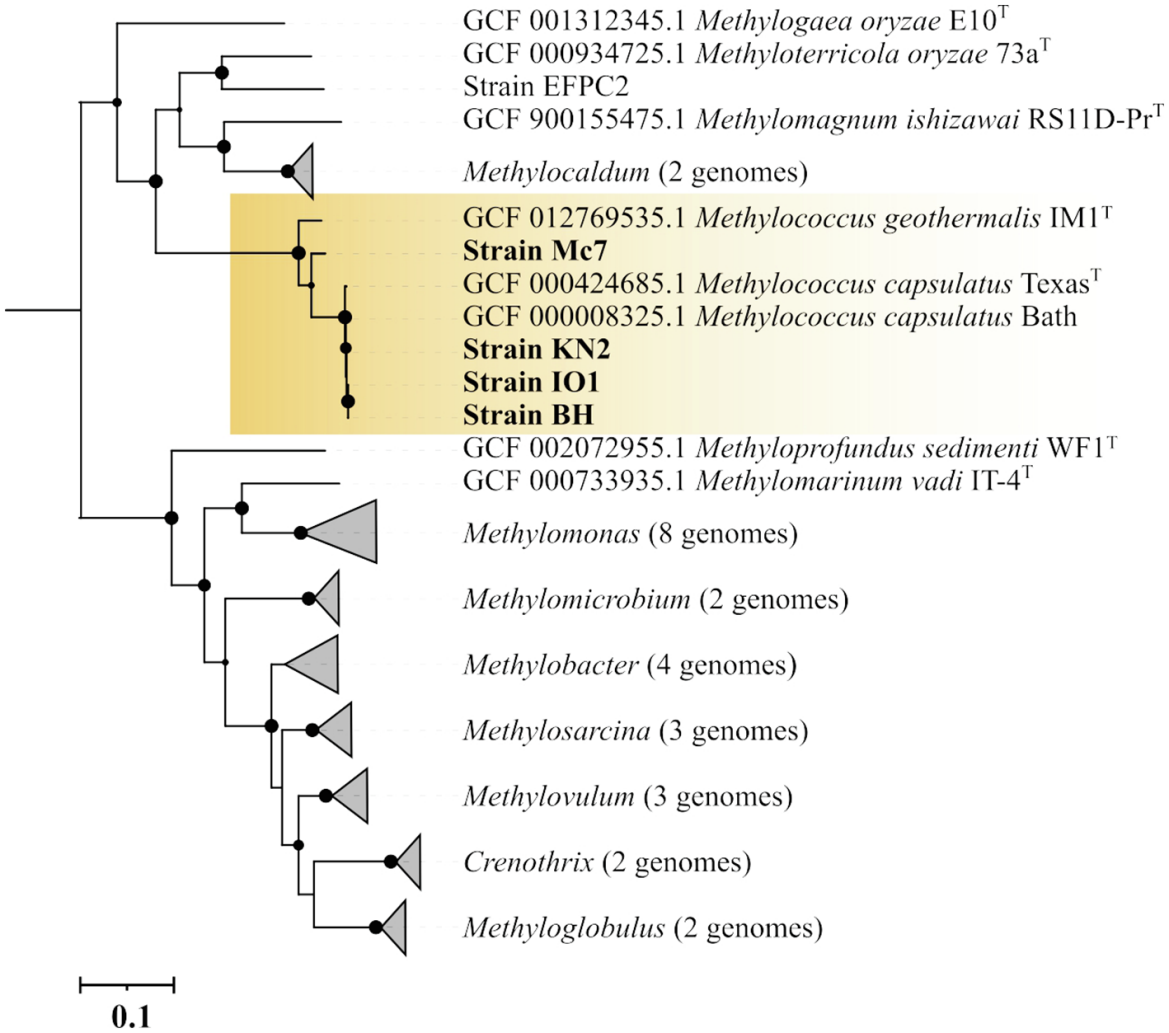
Genome-based phylogeny showing the position of new isolates in relation to other gammaproteobacterial methanotrophs based on the comparative sequence analysis of 120 ubiquitous single-copy proteins. The clade of *Methylococcus* methanotrophs is highlighted by orange. The tree was constructed using the Genome Taxonomy Database toolkit (Parks et al., 2018), release 04-RS89. The significance levels of interior branch points obtained in maximum-likelihood analysis were determined by bootstrap analysis (100 data re-samplings). Bootstrap values of >70% are shown. The root (not shown) is composed of all genomes available in GTDB for methanotrophs of the genera *Methylosinus* and *Methylocystis*. Bar, 0.1 substitutions per amino acid position.

The genome-based phylogeny of four novel *Methylococcus*-affiliated isolates was determined using the comparative sequence analysis of 120 ubiquitous single-copy proteins (Figure 3). Three isolates (strains IO1, KN2, BH) were clustered together with two strains of *M. capsulatus*, Texas^T^, and Bath, whereas strain Mc7 and *M. geothermalis* IM1^T^ constituted two separate branches within the genus *Methylococcus*. The complete genome of strain EFPC2, which is annotated in the GenBank as belonging to *Methylococcus* species, was also included in the analysis. However, as seen from Figure 3, strain EFPC2 clustered together with *Methyloterricola oryzae* 73a^T^ and did not affiliate with *Methylococcus* clade. The genome of strain EFPC2, therefore, was excluded from further comparative analysis of finished genomes available for *Methylococcus* species.

The average nucleotide identity (ANI) values were estimated for each pair of genomes (Supplementary Figure S2). ANI values calculated for isolates IO, KN2, BH, and *M. capsulatus* Bath were within a range of 98.75–99.73% which indicates that these organisms belong to the same species as intra-species level is defined at ≥95% ANI (Konstantinidis and Tiedje, 2005; Goris et al., 2007). ANI value determined for separately clustered strain Mc7 and *M. geothermalis* IM1^T^ was 88.56%. Thus, strain Mc7 may potentially represent a novel species of the genus *Methylococcus*.

**Figure 4:**
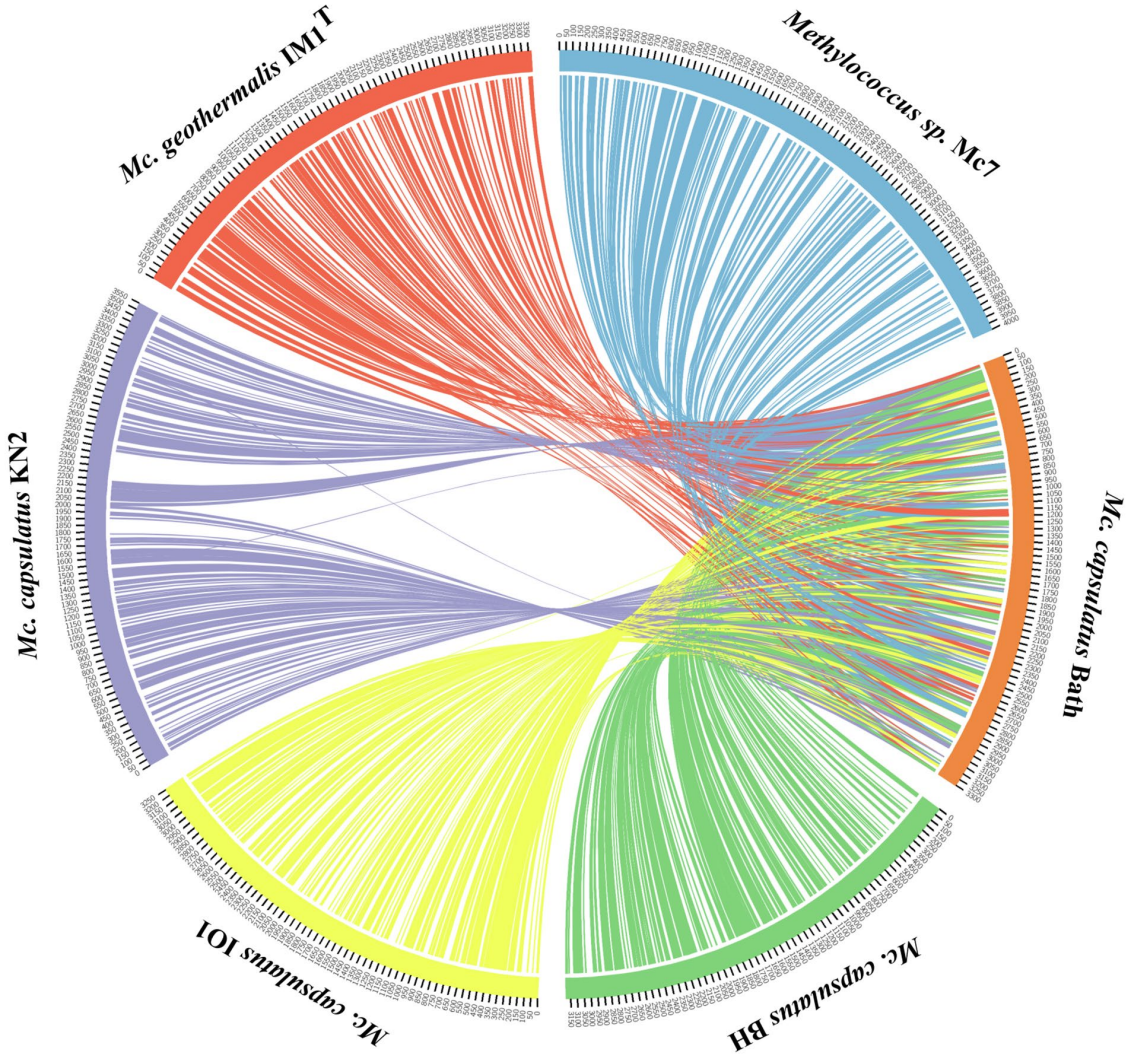
Visualization of synteny blocks between *Methylococcus capsualtus* Bath and other examined *Methylococcus* genomes. Each link represents a single synteny block. Circle is divided into the individual arcs representing genomes of *Methylococcus capsulatus* Bath (orange), *Methylococcus capsulatus* BH (green), *Methylococcus capsulatus* IO1 (yellow), *Methylococcus capsulatus* KN2 (purple), *Methylococcus geothermalis* IM1^T^ (red), and *Methylococcus* sp. Mc7 (blue).

The genomes of novel isolates, *M. capsulatus* Bath and *M. geothermalis* IM1^T^, were included in the whole-genome synteny analysis in order to explore the evolution of genome structure. Overall, 216 shared synteny regions were identified. Among those, 86 regions were common for all six genomes. Figure 4 depicts only the synteny blocks affiliated with *M. capsulatus* Bath.

**Figure 5:**
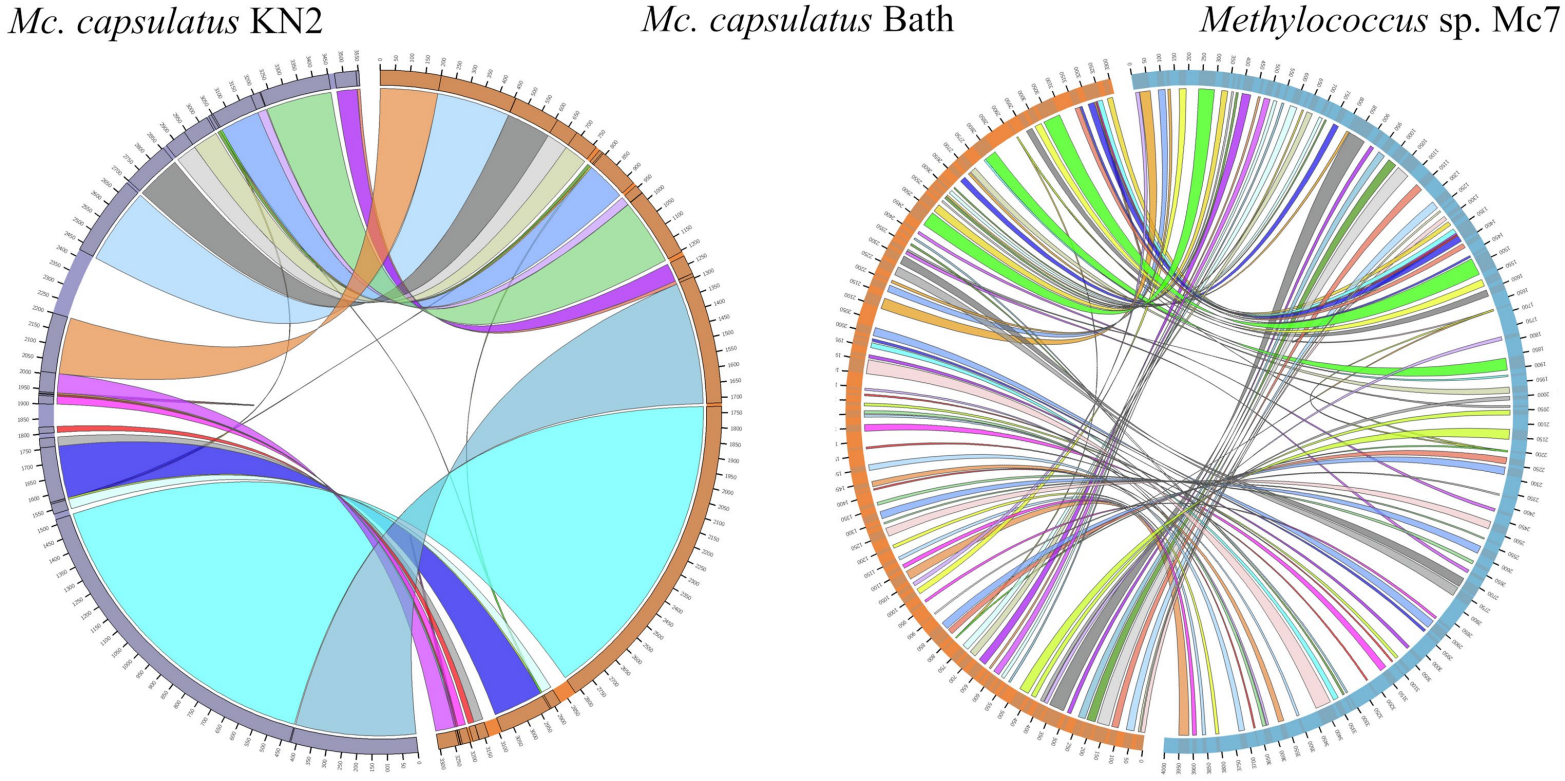
Visualization of synteny blocks between *Methylococcus capsulatus* Bath, *Methylococcus capsualtus* KN2, and *Methylococcus* sp. Mc7. Each link represents a single synteny block. Each circle is divided into two parts, which represent two compared genomes. The genome of *Methylococcus capsulatus* Bath is shown in orange, while the genomes of *Methylococcus capsulatus* KN2 and *Methylococcus* sp. Mc7 are displayed in purple and blue, respectively.

The number of synteny blocks revealed between *M. capsulatus* Bath and each of the isolates IO1, KN2, and BH was 102–103. Strain Mc7 and *M. geothermalis* IM1^T^ exhibited slightly lower levels of synteny to *M. capsulatus* Bath by displaying 97 and 93 synteny blocks, respectively. One of *M. capsulatus*-related isolates, strain KN2, and *Methylococcus* sp. Mc7 were chosen for more detailed analysis of whole-genome synteny patterns (Figure 5). As expected, the calculations made for two genomes resulted in lower number of synteny blocks, which were larger in size. Synteny regions shared between strain KN2 and *M. capsulatus* Bath covered 88.9 and 94.9% genomes of these bacteria, respectively. The largest synteny block in the genome of strain KN2 spanned about 1.1Mb and contained 978 genes. Two relatively large synteny break regions in the genome of strain KN2 spanned 224 and 76kb. Synteny breaks are caused by rearrangements, the insertion of novel genes, or the presence of genes that are too diverged to establish an orthologous relationship or have undergone expansion or loss (Liu et al., 2018a). Of 224 genes in the first synteny break region, 187 genes encoded hypothetical proteins. This region also included several genes coding for transposases, formate dehydrogenase displaying 95.5% amino acid sequence identity to NAD-dependent formate dehydrogenase from *Methylocaldum marinum*, flagellar transcriptional regulator FlhC, lysozyme RrrD, several helicases, and endonucleases. Of 84 genes in the second synteny break region in the genome of strain KN2, 76 genes encoded hypothetical proteins, while others coded for transport proteins. Comparison of the genomes of strain Mc7 and *M. capsulatus* Bath revealed 81 synteny blocks, which corresponded to nearly 50% of their genome length (Figure 5). The largest synteny break regions spanned 98.9, 98.5, 79.7, and 74.4kb. Approximately half of the genes within these regions encoded hypothetical proteins. Interestingly, 98.9kb region contained genes encoding small and large subunits of ribulose bisphosphate carboxylase.

### Pan-Genome Analysis

**Figure 6:**
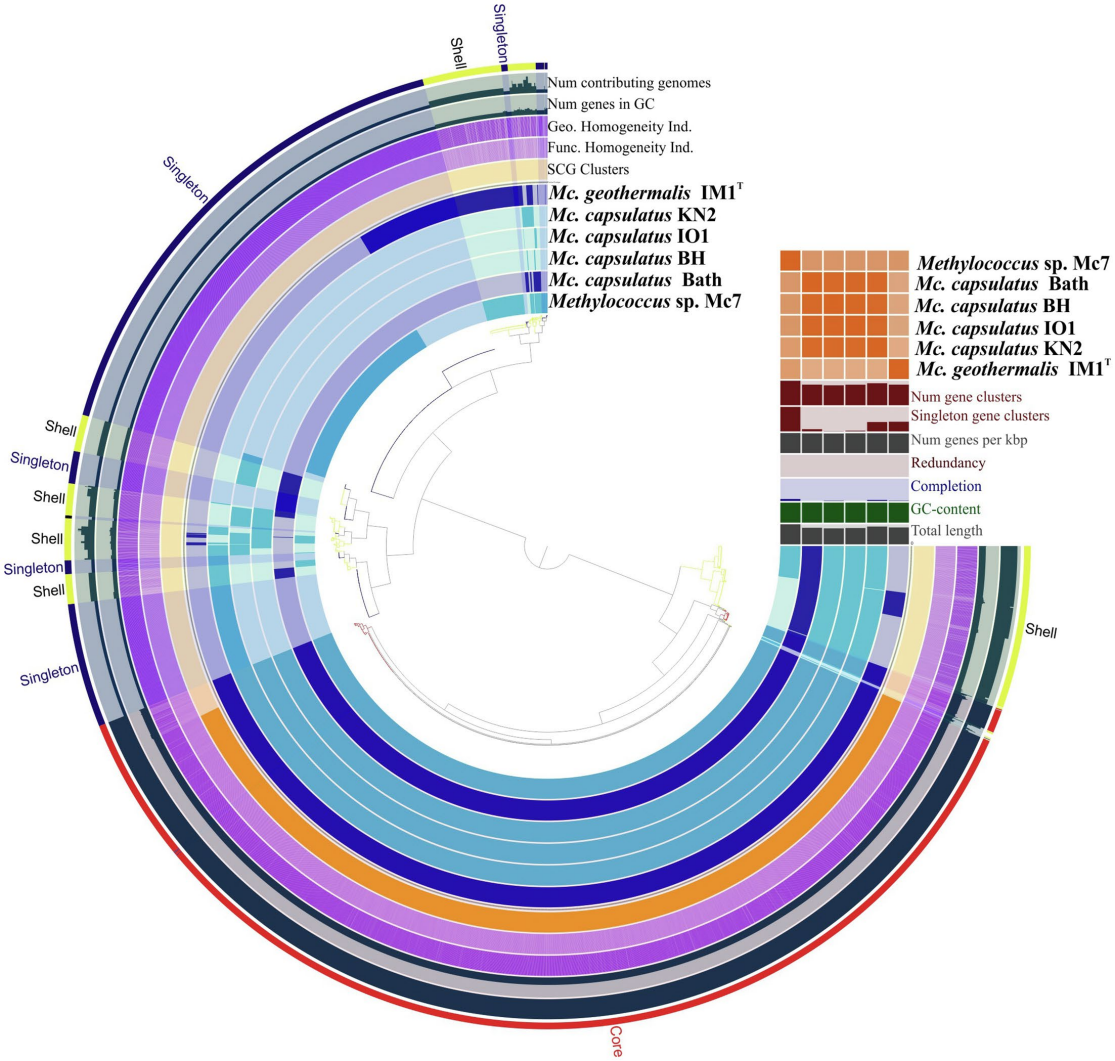
Pan-genome analysis. Clustering of genomes based on the presence/absence patterns of 4,485 pan-genomic clusters. The genomes are organized in radial layers as core, singleton, and shell gene clusters (Euclidean distance; Ward linkage) which are defined by the gene tree in the center. Genomes of new isolates are colored by blue. Genomes of *Methylococcus capsulatus* Bath and *Methylococcus geothermalis* IM1^T^ are colored by dark blue. Line segments indicate single gene clusters. The outermost circles carry the information regarding the number of genomes containing specific gene cluster, number of genes in gene clusters (gray), functional, geometric, and combined homogeneity indexes (purple), distribution of single-copy gene clusters (orange circle). The layers below the heatmap display from the bottom to top: total length of the genomes, GC content, genomes completion, redundancy of each genome, number of genes per kbp, number of singleton gene clusters, total number of gene clusters. Heatmap denotes correlation between the genomes based on average nucleotide identity values calculated using pyANI.

The Anvi’o pangenomics workflow was used to cluster protein-coding sequences into core, shell, and singleton genomes. Of 4,485 identified gene clusters, the *Methylococcus* pan-genome core comprised 2,331 genes (on average 51.9% of each genome), with the shell genome containing 846 gene clusters (18.9% of total gene clusters) and the cloud containing 1,308 gene clusters (29.2% of total gene clusters; Figure 6).

Pan-genome of the genus *Methylococcus* is open. Supplementary Figure S3 displays the number of core genes (A) and the pan-genome size (B) as a function of the number of included genomes. Fitting the curve in Supplementary Figure S3B to a power law (3,146.3*n*^0.171^) allows extrapolating calculations to *n* genomes. According to approximation, sequencing of one additional genome would provide 100 new genes to pan-genome. If the number of available genomes constituted 50 or 100 genomes, sequencing of one additional genome would contribute 21 and 12 genes, respectively. The core, shell, and cloud gene clusters were further annotated into COG classes (Supplementary Figure S4). The core genome was mostly conserved in the following: energy production and conversion (11.5% of total core gene clusters); translation, ribosomal structure, and biogenesis (8.6%); cell wall/membrane/envelope biogenesis (8.3%); coenzyme transport and metabolism (7.3%); inorganic ion transport and metabolism (6.3%); amino acid transport and metabolism (5.3%); posttranslational modification; protein turnover, chaperones (4.9%). However, the most abundant category was the one with no functional prediction (19.9%). The most abundantly represented functional categories in shell genome were replication, recombination, and repair, coenzyme transport and metabolism, signal transduction mechanisms, transcription, inorganic ion transport and metabolism, cell wall/membrane/envelope biogenesis, energy production and conversion. The majority of cloud genes were concentrated in the genomes of strain Mc7, *M. geothermalis* IM1^T^, and *M. capsulatus* KN2 and represented replication, transcription, cell wall/membrane/envelope biogenesis, and signal transduction mechanisms. These functional categories were also affiliated with few cloud genes found in *M. capsulatus* Bath and strain BH. A number of studies demonstrated that core genes systematically represented the smallest fraction of pan-genome (Bazinet, 2017; Livingstone et al., 2018; Kumar et al., 2020; Oshkin et al., 2020). The large proportion of core genes in *Methylococcus* pan-genome may be explained by both the small genome size of individual *Methylococcus* strains and the low number of available genomes that could be taken for analysis. However, approximation for core- and pan-genome (Supplementary Figure S3) suggests that even if 30 genomes were included into pan-genome analysis, the fraction of core genes would still be high (41.1% of the total gene clusters).

### Methane Oxidation Enzymes

Two complete copies of the *pmoCAB* gene cluster encoding pMMO and one additional copy of the *pmoC* (*pmoC3*) gene were revealed in the genomes of all novel *Methylococcus* strains. The corresponding *pmoA* sequences were nearly identical to each other and to the *pmoA* sequences from *M. capsulatus* Bath and *M. geothermalis* IM1^T^. The sMMO-encoding gene cluster *mmoXYBZDC* was also present in all examined genomes, with MmoX sequences exhibiting high level of homology (100% identity between MmoX from *M. capsulatus* Bath and strains KN2, IO1, BH, and 97% identity with that from strain Mc7). This chromosomal locus in *M. capsulatus* Bath and strain IO1 contained also one gene encoding a hypothetic protein between *mmoB* and *mmoZ* genes. The gene cluster *mmoGQSR* coding for the large subunit of bacterial chaperonin GroEL (*mmoG*), the two-component sensory/regulatory system (*mmoQ* and *mmoS*), and the transcription activator (*mmoR*; Csáki et al., 2003) was found in all examined genomes.

Two PQQ-dependent MDHs catalyze the second stage of C_1_-oxidation. The structural components of heterodimeric Ca^2+^-dependent MDH (MxaFI type) and the proteins required for its catalytic activity are encoded by the *mxaFJGIRACKLD* cluster with gene organization completely identical in all *Methylococcus* strains. Namely, the *mxaG* gene encodes the specific electron acceptor cytochrome c_L_, the *mxaACKL* gene cluster encodes the proteins required for Ca^2+^ incorporation into the active site, while the *mxaRS* gene cluster encodes two proteins of unknown functions (Chistoserdova, 2011; Vuilleumier et al., 2012). The four strains harbor gene cluster *xoxFJ*, which encodes an alternative MDH (XoxF) containing a rare earth element in the active site (Hibi et al., 2011), and a periplasmic solute-binding protein (XoxJ). No other genes relevant to methanol oxidation were present in this locus. The *xoxG* gene coding for a putative electron acceptor from XoxF and being only distantly related to the *mxaG* gene (Keltjens et al., 2014; Yu et al., 2017; Zheng et al., 2018) was found in all genomes and was located separately from *xoxFJ*.

Oxidative transformations of formaldehyde are mediated by the enzymes of the tetrahydrofolate (THF)-based pathway whose amino acid sequences in the novel isolates are almost identical to those in *M. capsulatus* Bath. The complete sets of genes for the tetrahydromethanopterin (THMP)-dependent pathway of formaldehyde oxidation are present in all *Methylococcus* genomes. Each strain possesses the three-subunit formate dehydrogenase, which catalyzes the last stage of methane oxidation, and a five-gene operon encoding the cytoplasmic NAD^+^-dependent formate dehydrogenase composed of (*abc*)_2_ subunits that catalyze the reversible reaction of formate oxidation to CO_2_ (Hartmann and Leimkühler, 2013). All strains of *M. capsulatus* (Bath, KN2, IO1, and BH) possess also the two-subunit formate dehydrogenase, whereas this enzyme is missing in strain Mc7 and *M. geotermalis* IM1^T^. The genomes of three strains, Mc7, KN2, and *M. geothermalis* IM1^T^, encode the one-subunit formate dehydrogenase, which is not present in *M. capsulatus* Bath, IO1 and BH. Thus, strain KN2 possesses four formate dehydrogenases in comparison with other *Methylococcus* strains possessing three orthologs each. Since no other evident differences in the arrays of C1 oxidizing enzymes of these strains were found, one may hypothesize that the extended array of formate dehydrogenases helps increasing removal of C1 metabolites *via* their oxidation to CO_2_. However, the exact role of different formate dehydrogenases in metabolism of methanotrophic bacteria remains to be elucidated.

### Carbon Assimilation Pathways

#### Ribulose Monophosphate Cycle

Genes encoding enzymes of the RuMP cycle are linearly duplicated in all studied *Methylococcus* genomes. The central part of this duplicated DNA locus (~5,000bp) contains the transaldolase gene, which is surrounded by the genes of phosphohexulose isomerase (*phi*), hexulose phosphate synthase (*hps*), fructose bisphosphate aldolase and transketolase (Ward et al., 2004). Each of the studied strains has a gene encoding the fusion protein hexulose phosphate synthase/isomerase. Further research is needed to clarify if this gene multiplicity for the RuMP cycle affects growth characteristics of methanotrophs.

#### Serine Pathway

All examined *Methylococcus* genomes contain the *sga–hpr–gck3* gene cluster coding for the serine-glyoxylate aminotransferase, hydroxypyruvate reductase, and 3-glycerate kinase, which are the key enzymes of the serine pathway. They also harbor genes for malyl-CoA lyase and malate thiokinase responsible for glyoxylate biosynthesis but lack PEP carboxylase. Pyruvate carboxylase that can fulfill the function of replenishing C_4_ TCA intermediates is present only in strain Mc7. Although this function can be assigned to oxaloacetate decarboxylase (Ward et al., 2004) or to malic enzyme, these enzymes catalyze predominantly irreversible decarboxylation reactions (Rozova et al., 2019; Xu et al., 2020). The gene for PPi-dependent PEP carboxykinase was not detected in the examined *Methylococcus* genomes. The absence of genes coding for any of the currently known C_3_ carboxylation enzymes in most *Methylococcus* representatives requires further efforts to decipher mechanisms for replenishing TCA cycle intermediates. In addition, glyoxylate can be generated from phosphoglycolate, which is the product of the oxygenase reaction of ribulose bisphosphate carboxylase (RuBisCo) as previously evidenced for *M. capsulatus* Bath (Taylor et al., 1981; Baxter et al., 2002). The genes for phosphoglycolate phosphatase and glycolate oxidase are present in the genomes of all *Methylococcus* representatives.

#### Calvin Cycle

Genomes of all *Methylococcus* strains possess *cbbL* and *cbbS* genes for the large and the small subunits of RuBisCo, as well as the *cbbQ* gene encoding a polypeptide putatively acting as a posttranslational RuBisCo activator (Baxter et al., 2002). Additional copies of these genes are present only in the genome of strain Mc7. Six genes encoding carbonic anhydrases were found in strain KN2, five genes in other *M. capsulatus* strains, and only three enzymes are encoded by Mc7 and IM1. Recent study suggests that RuBisCO is essential for *M. capsulatus* Bath growth and central metabolites derived from CO_2_ enter core intermediary metabolic pathways, including the Embden–Meyerhof–Parnas (EMP) glycolytic pathway, the pentose phosphate pathway, and the TCA cycle (Henard et al., 2021).

#### Central Metabolism

Several pathways for C_6_-phosphosugars degradation are encoded in the genomes of four novel *Methylococcus* isolates. These include the modified Embden–Meyerhof–Parnas pathway, where PPi-dependent phosphofructokinase catalyzes the reaction of fructose-1,6-phosphate synthesis from fructose-6-phosphate (Reshetnikov et al., 2008), the Entner–Doudoroff pathway, and the phosphoketolase pathway. The genomes of strain Mc7 and *M. geothermalis* IM1^T^ encode four isozymes of glucose-6-phosphate dehydrogenase, while all strains of *M. capsulatus* (Bath, KN2, IO1, BH) possess only two of these isozymes. The genomes of novel isolates encode phosphoketolase catalyzing cleavage of fructose-6-phosphate or xylulose-5-phosphate into glyceraldehyde-3-phosphate/erythrose-4-phosphate and acetyl phosphate; the genes coding for phosphoketolase and acetate kinase comprise a single cluster. These strains, therefore, are predicted to be able to convert acetylphosphate to acetate and to produce ATP as previously suggested for other gammaproteobacterial methanotrophs (Rozova et al., 2015; Henard et al., 2017). In strain Mc7 and *M. geothermalis* IM1^T^, acetyl phosphate can be directly converted into acetyl-CoA without ATP consumption, while strains of *M. capsulatus* lack the respective acetyl phosphotransferase. In all *Methylococcus* species, the synthesis of acetyl-CoA can also proceed from acetate by ATP-dependent acetyl-CoA synthetase forming AMP. Interestingly, two strains, KN2 and Bath, code for two enolases, while other strains possess only one enzyme. The relative excess of enolase, which directs the primary C3 intermediates of the Calvin cycle and the serine pathway to the central metabolism, most likely, can increase the impact of these “minor” pathways in overall carbon assimilation of these methanotrophs.

It is known that gammaproteobacterial methanotrophs are capable of fermentation under micro-oxic conditions (Kalyuzhnaya et al., 2013). The genomes of novel *Methylococcus* isolates contain the same genes potentially involved in fermentation (pyruvate formate lyase, alcohol dehydrogenase, acetate kinase) as *M. capsulatus* Bath (Ward et al., 2004). However, similar to strain Bath, these methanotrophs lack the lactate dehydrogenase gene.

#### Nitrogen Metabolism

All *Methylococcus* strains possess the structural genes for nitrogenase (*nifHDK*) apparently constituting an operon together with *nifENX*; the latter being involved in synthesis of the nitrogenase iron-molybdenum cofactor. One gene of unknown function is present between *nifK* and *nifE* in strains Bath, BH, KN2, and IO1, whereas the same locus in strain Mc7 contains three genes. All strains possess the gene cluster responsible for nitrate reduction, i.e., nitrate reductase (NasA), nitrite reductase (NirBD), protein kinase and nitrate transporter. All strains contain the genes *haoAB* for putative hydroxylamine dehydrogenase that transforms hydroxylamine to nitric oxide. One potential source of hydroxylamine is ammonium oxidation by pMMO since these methanotrophs lack ammonia monooxygenase. The gene cluster *norBC* responsible for reduction in nitric oxide to nitrous oxide is also present in the genomes. Like *M. capsulatus* Bath (Ward et al., 2004), novel strains encode three predicted hydrogenases: a multisubunit formate hydrogen lyase, most likely involved in the conversion of formate to dihydrogen and carbon dioxide; a soluble cytoplasmic NAD-reducing hydrogenase, which transfers electrons to NAD; and a membrane-bound Ni-Fe hydrogenase.

#### Secondary Metabolism

The genomes of novel isolates as well as the genome of *M. capsulatus* Bath encode enzymes of glycogen synthesis, i.e., 4-α-glucanotransferase (amylomaltase), glucose-1-phosphate adenylyltransferase, 1,4-α-glucan-branching enzyme GlgB, and glycogen synthase. The corresponding genes are organized in one cluster similar to that in *M. capsulatus* Bath. All strains, including *M. capsulatus* Bath, also harbor an additional gene cluster, which encodes glycogen synthase 2 and α-amylase. Among examined *Methylococcus* representatives, only strain Mc7 possesses genes coding for sucrose biosynthesis and degradation. The genes for sucrose phosphate synthase, sucrose phosphate phosphatase, and sucrose synthase are assembled in one gene cluster in strain Mc7 similar to that in *Methylocaldum szegediense* O12 (But et al., 2018). Like the latter, strain Mc7 possesses the gene coding for mannose-1-phosphate guanyltransferase; however, it lacks fructokinase encoding gene. The ability to synthesize sucrose may be one of the reasons behind the enhanced salt tolerance observed for strain Mc7 in comparison with other *Methylococcus* isolates (Table 1).

The genomes of the studied methanotrophs encode enzymes homologous (40–42% sequence identity) to the previously characterized acetolactate decarboxylase from *Klebsiella aerogenes* (Blomqvist et al., 1993). Interestingly, in *Methylococcus* species, the acetolactate decarboxylase gene forms a cluster together with genes of the malic enzyme and acetolactate synthase; the latter known to produce acetolactate from two molecules of pyruvate with CO_2_ release (Blomqvist et al., 1993). Given that acetolactate decarboxylase degrades acetolactate into acetoin and CO_2_, functioning of a fermentation pathway that converts malate to acetoin through pyruvate and acetolactate appears to be possible. The BLAST search revealed that, besides methanotrophs of the genus *Methylococcus*, acetolactate decarboxylase is also encoded in the genomes of *Methyloterricola oryzae*, *Methylogaea oryzae* as well as in *Methylovulum* and *Methyloprofundus* representatives. In microorganisms, the pathway of acetoin synthesis is regarded as mechanism preventing over-acidification of the intracellular environment, and also as an energy-storing strategy (Xiao and Xu, 2007).

**Figure 7:**
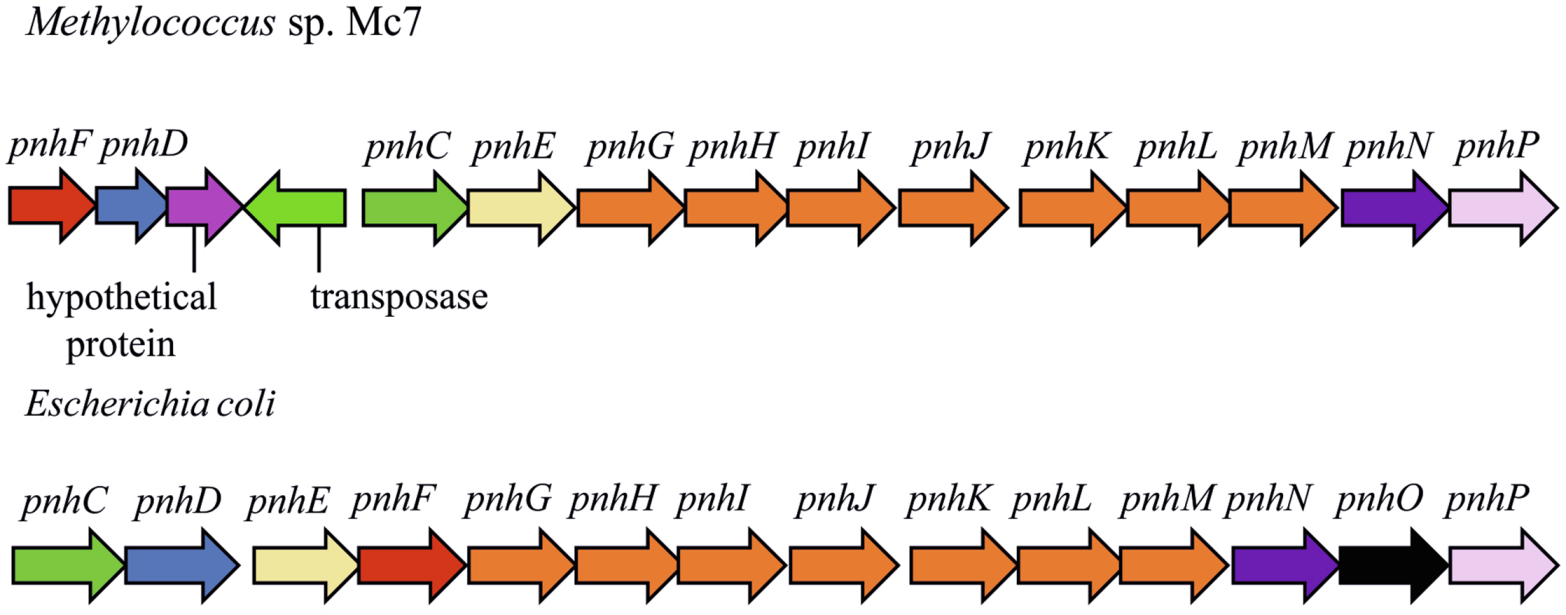
The gene clusters carrying genes of pathway for organophosphonates degradation in methylotrophs and *Escherichia coli*. The products of the *phn* genes were proposed based on the comparison with the respective sequences in the *Escherichia coli* operon (Jochimsen et al., 2011): phosphonate ABC transporter ATP binding subunit (*phnC*); phosphonate ABC transporter periplasmic binding protein (*phnD*); phosphonate transport system permease protein (*phnE*); putative transcriptional regulator (*phnF*); carbon-phosphorus lyase core complex subunits (*phnGHIJK*); methylphosphonate degradation complex subunit (*phnL*); RPnTP hydrolase (*phnM*); ribose-1,5-bisphosphate phosphokinase (*phnN*); aminoalkylphosphonate N-acetyltransferase (phnO); 5-phospho-alpha-D-ribosyl-1,2-cyclic phosphate phosphodiesterase (*phnP*).

In strain Mc7, DNA locus of ~14,000bp is represented by the 13-gene cluster *phnFFCEGHIJKLMNP*, which displays high structural and sequence similarity (27–54% amino acid sequence identity) to the *Escherichia coli* operon encoding the carbon-phosphorus (CP) lyase (Figure 7). In *E. coli*, this gene cluster was found to determine degradation of organophosphonates (Adams et al., 2008; Jochimsen et al., 2011) and we may assume the same function of the corresponding genes in strain Mc7. BLAST search revealed that, among gammaproteobacterial methanotrophs, these genes are present only in the genome of marine methanotroph *Methyloprofundus sedimenti* WF1^T^. Organophosphonates are widely distributed in nature and are represented by various naturally occurring compounds (phosphonopyruvate, 2-aminoethylphosphonate, and phosphonoacetate; Ternan et al., 1998; Seto and Kuzuyama, 1999) as well as by synthetic xenobiotics such as herbicide glyphosate ([N-(phosphonomethyl) glycine]). Obviously, the potential substrates of CP lyase from strain Mc7 and *M. sedimenti* WF1^T^ are naturally occurring organophosphonates. The ability to decompose a highly stable carbon-phosphorous bond allows survival in habitats with low contents of phosphates.

The genomes of strain Mc7 and *M. geothermalis* IM1^T^ contain the genes for haloalkane dehalogenase, which catalyzes the hydrolytic cleavage of carbon–halogen bonds (EC 3.8.1.5). It is absent in *M. capsulatus* strains, whereas its homologues were found in the genomes of *Methylocaldum* spp. (with ~80% identity), *Methylobacter tundripaludum* and *Methylovulum myaconense* (<70%) as well as in alphaproteobacterial methanotrophs (<50%). The corresponding protein shares 51% identity with haloalkane dehalogenase characterized from *Bradyrhizobium japonicum* (Sato et al., 2005).

The genome of strain Mc7 contains the gene for putative methanethiol S-methyltransferase, which catalyzes the transmethylation between methanethiol (MeSH) and S-adenosyl-l-methionine into dimethylsulfide (DMS) and S-adenosyl-l-homocysteine as well as the genes for FMNH_2_-dependent dimethylsulfone monooxygenase and FMNH_2_-dependent monooxygenase SfnG. MeSH can be formed by sulfide methylation in anaerobic environments or by degradation of sulfur-containing amino acids, *via* the cleavage of dimethylsulfopropionate, which is osmoprotector in marine microalgae, or by demethiolation of sulfhydryl groups (Lomans et al., 2001, 2002; Bentley and Chasteen, 2004). Further metabolism of DMS can proceed *via* chemical or biochemical oxidation into dimethylsulfone. The latter compound, being substrate for FMNH2-dependent monooxygenases, is converted into inorganic sulfite and formaldehyde. Obviously, this mechanism of scavenging sulfur from organosulfur compounds enables bacterial survival in sulfate-depleted environments (Boden et al., 2011; Carrión et al., 2015). All strains under study possess genes for the sulfur oxidizing (Sox) system that allows utilization of inorganic sulfur compounds in energy metabolism. In these methanotrophs, the Sox system is represented by the SoxYZ complex that presumably carries the intermediates of the pathway on a cysteine residue near the C terminus of SoxY as well as by sulfane dehydrogenase SoxCD; the latter has been found to catalyze a six-electron oxidation reaction (Friedrich et al., 2005). However, all *Methylococcus* strains lack SoxB, SoxA, and SoxX components needed for the full Sox system. Functionality and role of the truncated pathway of sulfur oxidative metabolism in the methanotrophs is not clear.

### Integrons

Integrons are gene-capturing platforms that play a significant role in generating phenotypic diversity and shaping adaptive responses in microorganisms, including the spread of antibiotic resistance genes (Mazel, 2006; Gillings, 2014). The integron structure includes variable gene cassette array (recombination site attC with corresponding genes) and a stable platform containing integrase (IntI), recombination site (attI) and promoter (Boucher et al., 2007). Of all novel *Methylococcus* isolates, integron-like elements were detected only in the genome of strain Mc7. This strain contains one complete integron with one attC site, one separate attC site, large attC-cassette (nine sites), and stand-alone integrase. The gene encoding 6-phosphogluconate phosphatase was found in a large attC-cassette; the functions of other genes near attC sites could not be predicted. A complete integron containing three attC sites was also revealed in the genome of *M. geothermalis* IM1^T^.

### Integrative and Conjugative Elements/Integrated Mobilizable Elements

**Figure 8:**
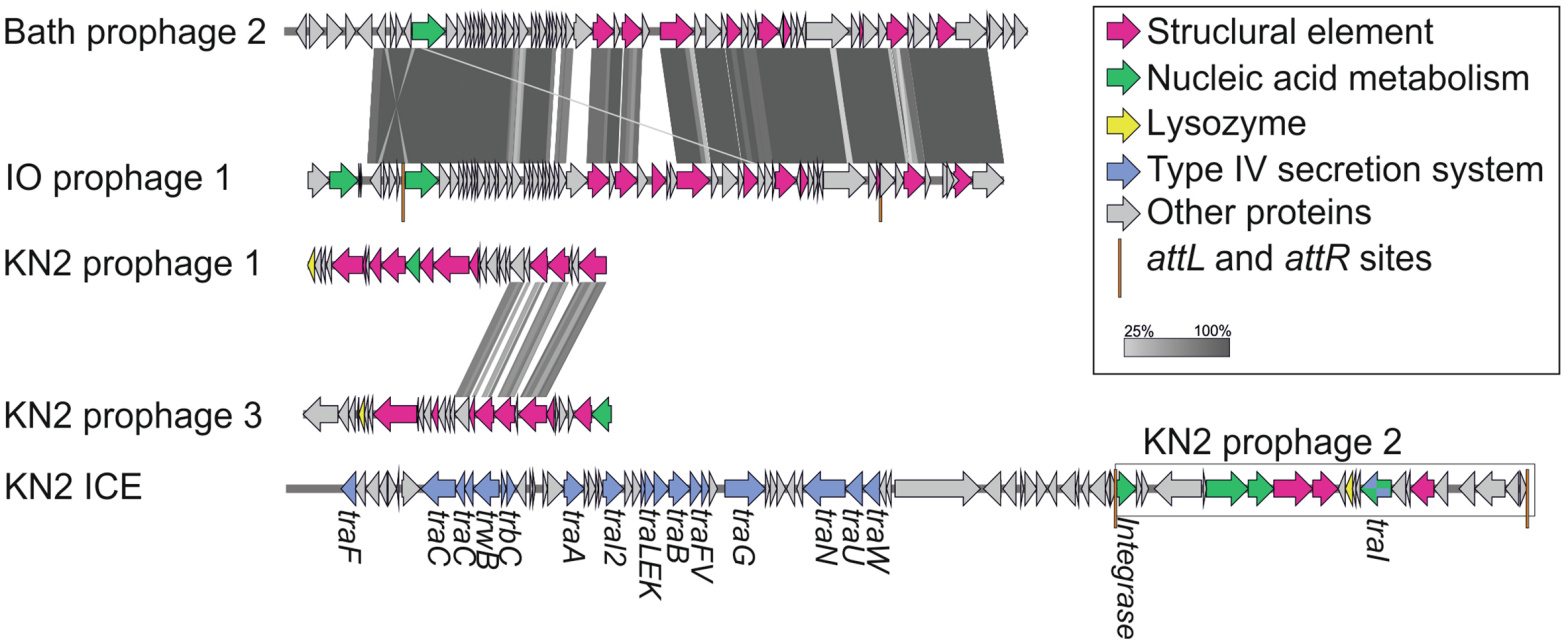
The structure of integrative and conjugative element (ICE) region revealed in the genome of strain KN2 and prophage-associated regions (≥20Kb) detected in the examined *Methylococcus* genomes.

Integrative and conjugative elements (ICEs) are widespread mobile DNA that transmit both vertically, in a host-integrated state, and horizontally, through excision and transfer to new recipients. Recent findings indicated that the main actors of conjugative transfer are not the well- known conjugative or mobilizable plasmids but the integrated mobilizable elements (IMEs; Delavat et al., 2017; Guédon et al., 2017). IMEs encode their own excision and integration and use the conjugation machinery of unrelated co-resident conjugative element for their own transfer (Guédon et al., 2017). One putative IME was found in the genome of strain Mc7; unfortunately, the ends of the element could not be identified. A putative IСE region of 123,456bp was revealed in the genome of strain KN2. This region contained the genes of the type IV secretion system, which plays a key role in conjugation (Lawley et al., 2003). Interestingly, some fragments of a prophage were also found in this ICE region (Figure 8).

### Phages

One to six prophage regions were found in the genomes of the examined *Methylococcus* strains (Table 3). Potentially complete prophages were detected in the genomes of strains IO1 and KN2. In the prophage region of strain IO1, the attL and attR sites were displaced, and the lysozyme genes were also absent (Figure 7). This prophage is structurally highly similar to the prophage 2 from *M. capsulatus* Bath. The prophage region in the genome of strain KN2 was, most likely, divided into large fragments as a result of integrase activity. This suggests that the conversion of these prophages to the lytic cycle is unlikely.

**Table 3:** Prophage regions revealed in the examined *Methylococcus* genomes.

Strain	Region no.	Region length, kb	Region position	tRNA	Total proteins	Number of phage protein hits	att_site present
Bath	1	36.1	2,819,640–2,855,795	0	40	20	No
	2	51.7	3,091,049–3,142,838	0	58	44	Yes
IO1	1	48.4	2,508,110–2,556,599	2	64	45	Yes
KN2	1	20.8	1,847,198–1,868,022	0	26	19	No
	2	29	2,258,604–2,287,649	0	18	12	Yes
	3	21.4	2,313,376–2,334,848	0	27	16	No
	4	15.9	3,029,181–3,045,103	0	21	12	No
Mc7	1	13	1,444,044–1,457,136	0	8	6	Yes
	2	7.4	3,652,375–3,659,823	0	7	6	No
IM1^T^	1	11.6	672,463–684,108	1	12	7	No
	2	11.3	2,433,262–2,444,593	0	8	7	No
BH	1	11.3	501,048–512,403	0	8	7	No
	2	7.8	650,531–658,368	0	8	6	No
	3	8.5	1,088,481–1,096,982	0	10	6	No
	4	4.1	1,607,407–1,611,579	0	8	7	No
	5	7.7	2,024,931–2,032,721	0	9	6	No
	6	8.1	2,164,268–2,172,463	0	8	6	No

### Insertion Sequences

**Figure 9:**
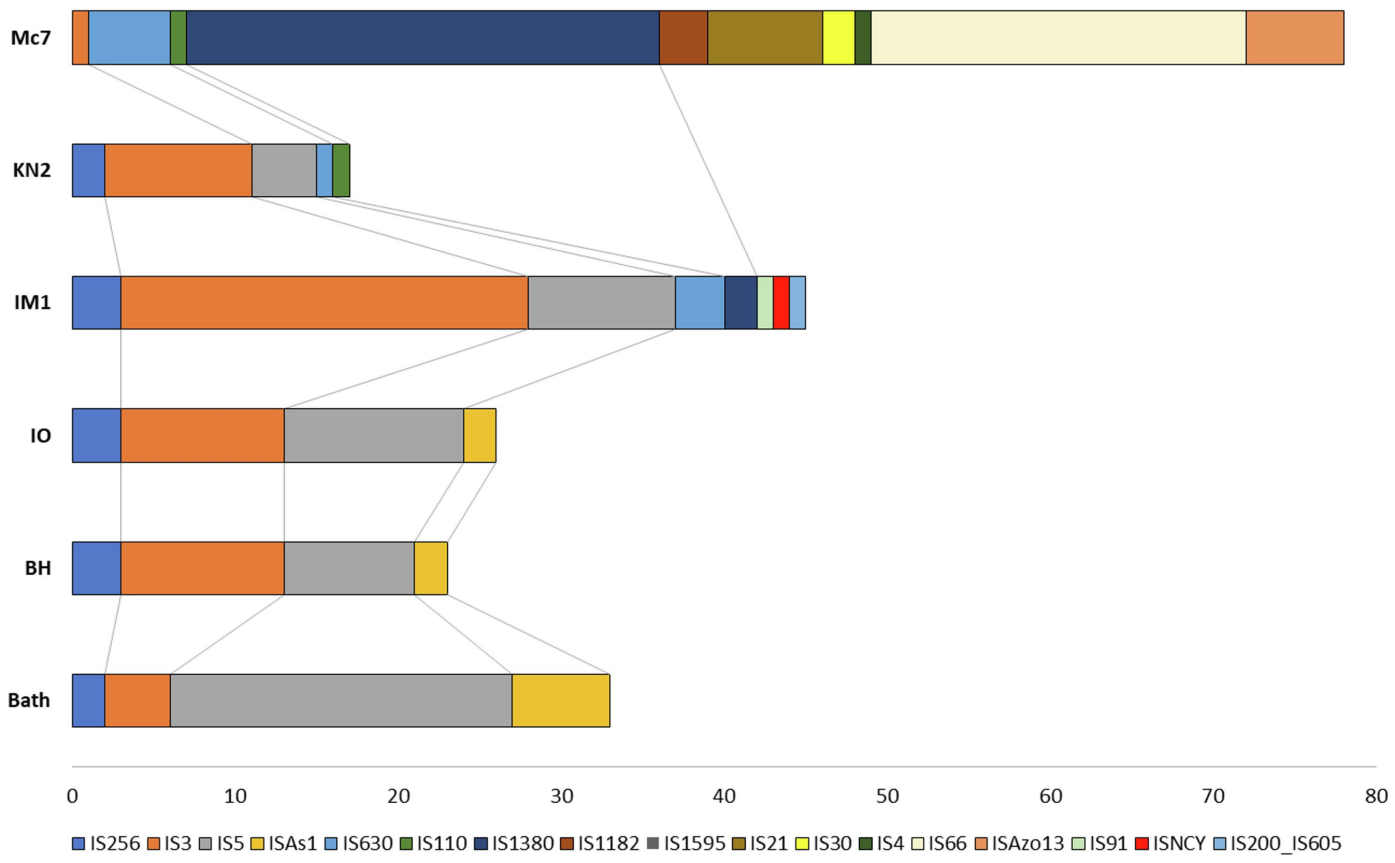
Distribution of insertion sequences (IS) element families in *Methylococcus* genomes.

Insertion sequences are transposable DNA segments ranging in length from 0.7 to 3.5kb, generally including a transposase gene encoding the enzyme that catalyzes IS movement. The number of complete IS in the genomes of studied strains varied from 17 in KN2 to 78 in Mc7 (Figure 9). Similar to *M. capsulatus* Bath, the genomes of strains IO1 and BH contained IS elements belonging to IS256, IS3, IS5, and ISAs1 families (Siguier et al., 2014). The total number of complete IS elements in these three strains was also similar. By contrast, the genomes of strains IO1 and IM1^T^ did not contain IS elements of the ISAs1 family but contained one IS of the IS110 and IS630 families. Strain IM1^T^ also contained IS of IS91, ISNCY, IS200_IS605 families, which were not revealed in other studied genomes. Among the studied strains, the Mc7 genome was noticeably distinguished by both the total number and the variety of IS elements. IS massive expansion is accompanied by gene inactivation and decay, genome rearrangement, and genome reduction (Siguier et al., 2014). Some IS elements, for example, IS30 (Dalrymple and Arber, 1985) are capable of reactivating the expression of nearby genes. Therefore, IS elements can play an important role in the adaptation of the host cell to new lifestyle, such as continuous cultivation for the needs of industry (Gaffé et al., 2011). High similarity of the IS family composition between strains Bath, IO1, and BH suggests low influence of a lateral gene transfer on the evolution of their genomes. The change in the IS composition in other strains may be associated with the activity of other mobile elements, i.e., integrons in the genomes of strains Mc7 and *M. geothermalis* IM1^T^, and ICE in the genome of strain KN2.

In summary, this study expanded the pool of phenotypically characterized *Methylococcus* strains with good-quality genome sequences by contributing four novel isolates of these bacteria from various habitats. Three novel isolates, strains KN2, IO1, and BH, were assigned to the species *M. capsulatus* and displayed surprisingly high similarity in gene content to that in *M. capsulatus* Bath. As evidenced by the original study of Foster and Davis (1966) and our work, methanotrophs of this species tend to inhabit nutrient-rich freshwater habitats, such as wastewater, sewage or sediments. They possess relatively small (3.2–3.6Mb) genomes and display high growth rates on methane in the temperature range between 42 and 52°C. The ability to grow on methanol, however, may vary in different members of this species. Thus, *M. capsulatus* Bath displayed only a trace growth on methanol. The same was true for one of the isolates obtained in our study, strain IO1, which showed poor growth only at low methanol concentrations (0.05–0.1%, *v*/*v*). By contrast, two other strains, KN2 and BH, were capable of growth in a wide range of methanol concentrations, up to 3.0% CH_3_OH in case of strain KN2. Notably, high resistance of strain KN2 to methanol correlates with the occurrence of four different isozymes of formate dehydrogenase in this methanotroph. This array of formate dehydrogenases may potentially be responsible for the efficient oxidation of С1 metabolites to CO_2_, thus determining high resistance to methanol. However, the exact role of different formate dehydrogenases in metabolism of methanotrophic bacteria remains to be clarified. Strain KN2 was also distinct with regard to its temperature optimum (48–52°C), which was higher than that in other isolates and strain Bath. Finally, strain KN2 showed the best performance during its continuous cultivation in a bioreactor. Taken together, *M. capsulatus* KN2 has the highest potential for the biotechnologies implying high growth rates on methane and/or abilities to grow on methanol.

The fourth isolate obtained in our study from a landfill cover soil, strain Mc7, may potentially represent a novel species of the genus *Methylococcus*. This is suggested by the results of phylogenomic analysis, comparative genome analysis as well as by the phenotypic difference of strain Mc7 and two previously described *Methylococcus* species. Thus, cells of strain Mc7 (~1.6μm in diameter) were larger that cells of *M. capsulatus* (1.0–1.1μm) or *M. geothermalis* (0.7–1.0μm; Awala et al., 2020). The genome size of strain Mc7 (4.0Mb) also exceeded that in other described *Methylococcus* species. Notably, the genome of this methanotroph from a landfill cover soil contained high number and variety of mobile elements, which may have played an important role in the adaptation of strain Mc7 to highly variable conditions of its natural habitat. Thus, strain Mc7 possessed a number of genome-encoded features, which were absent in strains of *M. capsulatus*, such as sucrose biosynthesis and the ability to scavenge phosphorus and sulfur from the environment. These metabolic capabilities may represent important components of the genome-determined environmental adaptations of this methanotroph. Further examination of phenotypic and chemotaxonomic features is needed to establish the taxonomic position of strain Mc7.

Thus, our study contributes to the current knowledge of *Methylococcus*-like methanotrophs, the bacteria of high environmental and biotechnological relevance. As suggested by the pangenome analysis, the metabolic diversity within this genus remains underestimated and calls for further cultivation- and genome-based studies.

## Data Availability Statement

The datasets presented in this study can be found in online repositories. The names of the repository/repositories and accession number(s) can be found at: https://www.ncbi.nlm.nih.gov/genbank/, CP079095–CP079098.

## Author Contributions

SD and NP obtained funding and designed the study. OD and IO isolated novel *Methylococcus* strains. IO obtained and annotated the genome sequences. IO, KM, SBu, and VK performed the comparative genome analysis. IO, RS, KM, ET, and SBe conducted physiology and growth tests. ET and NK assisted with experiments on continuous cultivation in bioreactor. IO, KM, VK, and SD wrote the manuscript. All authors contributed to the article and approved the submitted version.

## Funding

The article was made with support of the Ministry of Science and Higher Education of the Russian Federation in accordance with agreement no. 075-15-2020-907, date November 16, 2020 on providing a grant in the form of subsidies from the Federal budget of Russian Federation. The grant was provided for state support for the creation and development of a World-Class Scientific Center “Agrotechnologies for the Future”.

## Conflict of Interest

The authors declare that the research was conducted in the absence of any commercial or financial relationships that could be construed as a potential conflict of interest.

## Publisher’s Note

All claims expressed in this article are solely those of the authors and do not necessarily represent those of their affiliated organizations, or those of the publisher, the editors and the reviewers. Any product that may be evaluated in this article, or claim that may be made by its manufacturer, is not guaranteed or endorsed by the publisher.

## Footnotes

^1^ https://github.com/Ecogenomics/GtdbTk

^2^ http://phaster.ca/

^3^ http://biodev.cea.fr/virfam/
