# Supplementary material for: Expanding Characterized Diversity and the Pool of Complete Genome Sequences of Methylococcus Species, the Bacteria of High Environmental and Biotechnological Relevance
Source: Front Microbiol. 2021 Oct 6;12:756830. doi: 10.3389/fmicb.2021.756830 (PMC8527097; doi:10.3389/fmicb.2021.756830)
Supplement: Supplementary file 1 [file Data_Sheet_1.PDF]

Supplementary Material

Supplementary Table S1. Sequencing statistics.

	Characteristics	Strain KN2	Strain BH	Strain Mc7	Strain IO1
**Nanopore**	Number of reads	278437	107337	219674	192565
	Total bases, Gb	1.5	1	1.6	1.6
	N50	12996	19494	19494	17174
	Mean read length, bp	5555	9764	7233	8194
**Illumina**	Mean read length, bp	250	250	250	250
	Number of reads	415244	226980	3477384	573050

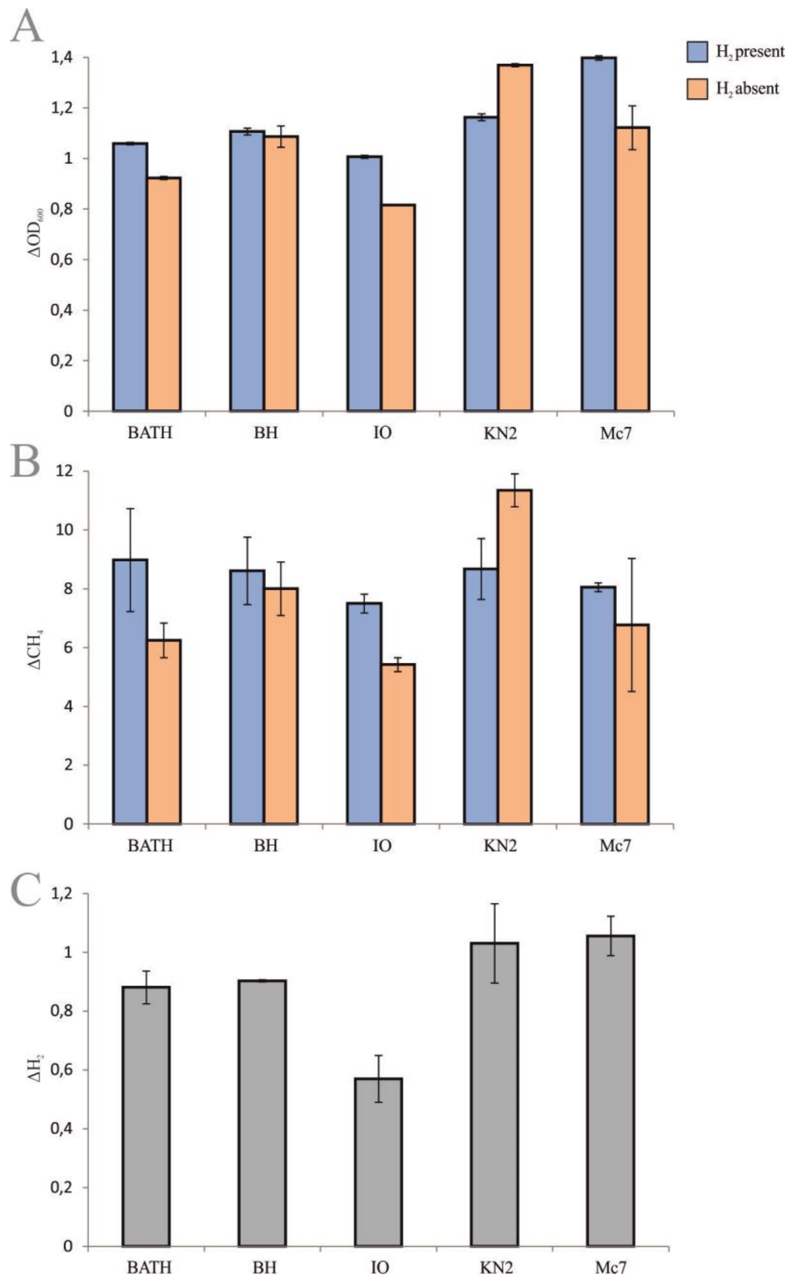

Supplementary Figure S1. Utilization of hydrogen by *Methylococcus* strains: (A) growth yield after 24 h of incubation with CH_4 and H_2 (blue) versus only CH_4 (orange); (B) methane consumption (%) registered after 24 h of incubation with CH_4 and H_2 (blue) versus only CH_4 (orange); (C) hydrogen consumption (%) detected in the presence of CH_4.

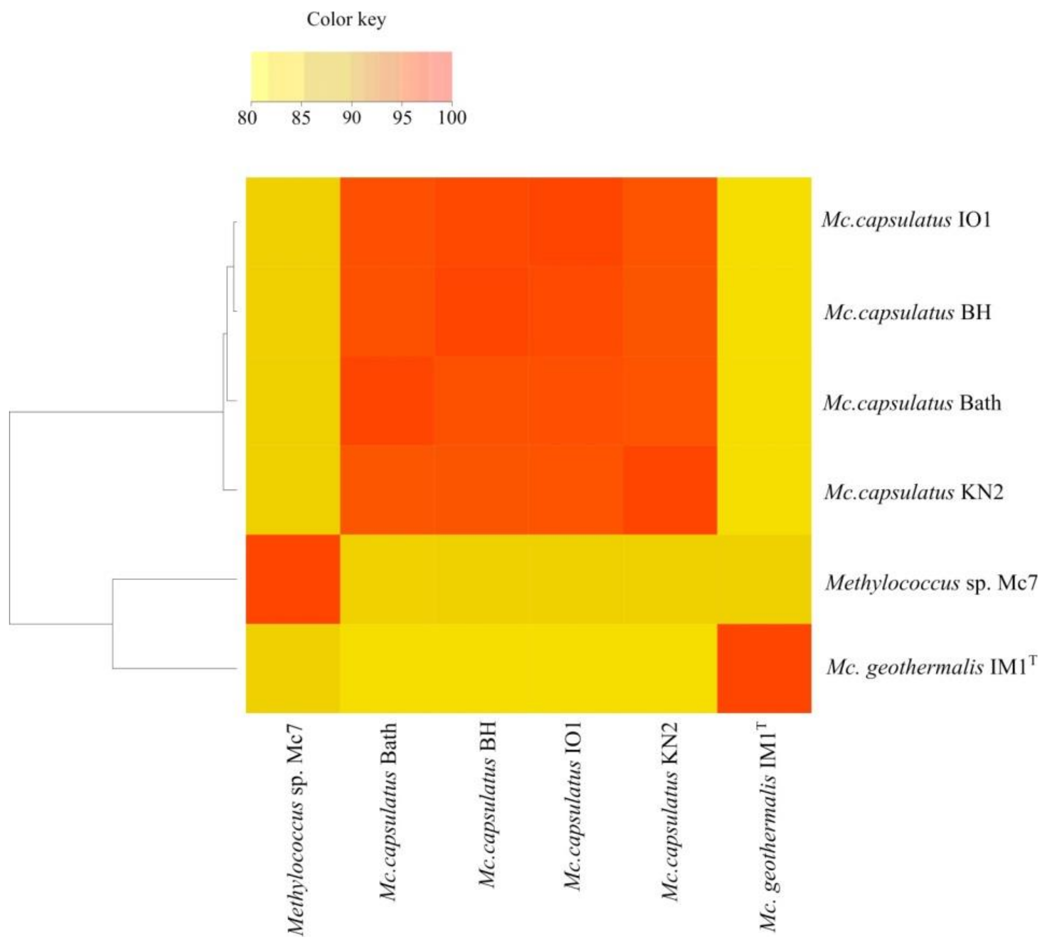

Supplementary Figure S2. Heatmap derived from an Average Nucleotide Identity (ANI) matrix calculated for each pair of the *Methylococcus* genomes examined in this study.

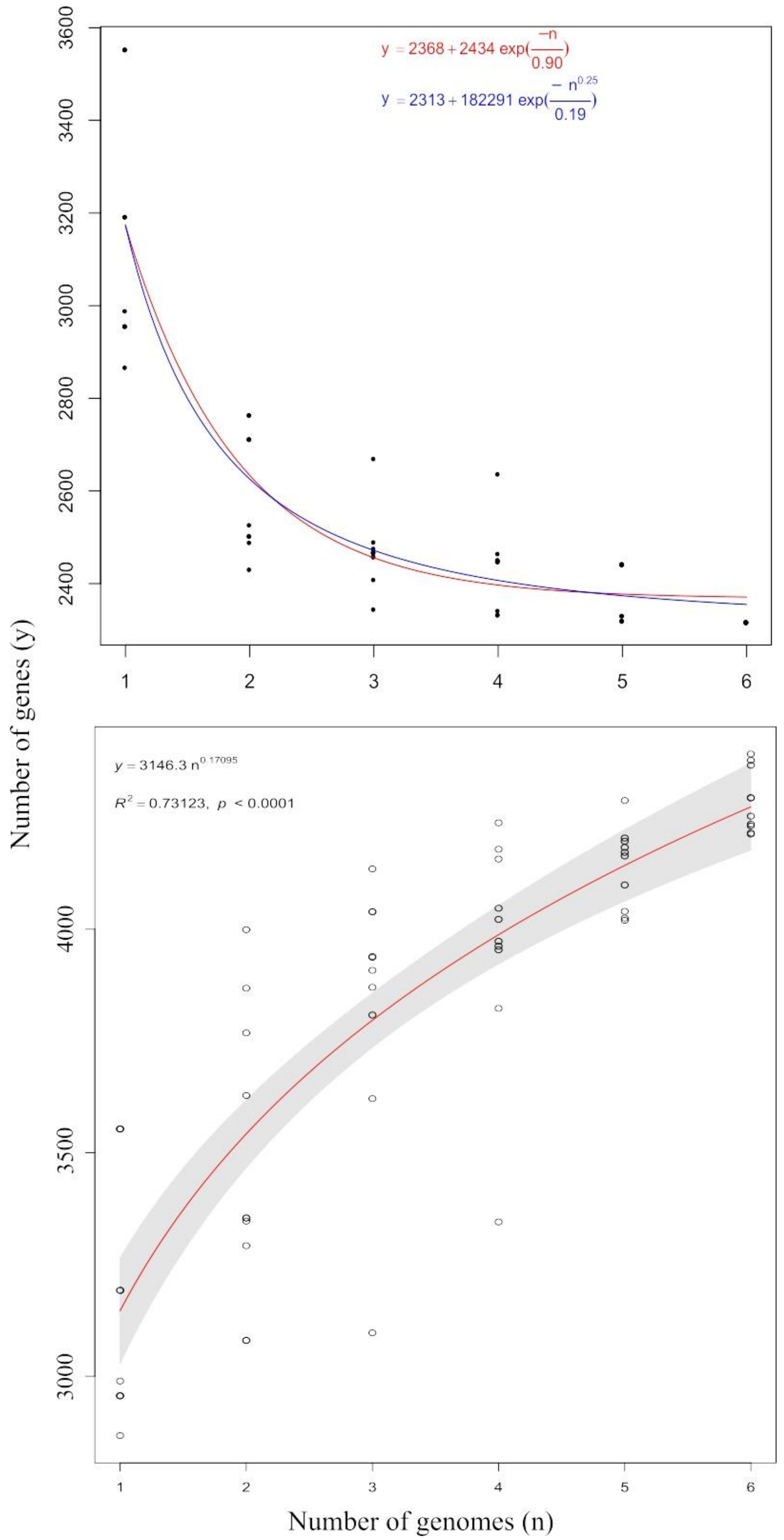

Supplementary Figure S3. The *Methylococcus* core genome (A) and pan-genome (B) as a function of the number of genomes included.

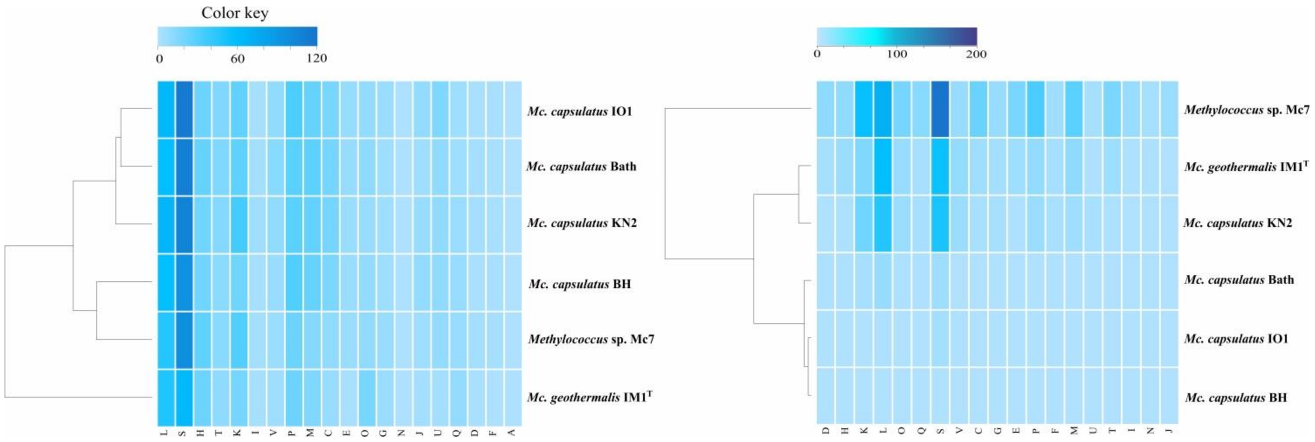

Supplementary Figure S4. Functional annotation of shell (left panel) and cloud (right panel) genes against COG database. The core genome is shared among all strains and, therefore, is not shown in this figure. Genes were automatically assigned to the following COG categories: A, RNA processing and modification; B, chromatin structure and dynamics; C, energy production and conversion; D, cell cycle control and mitosis; E, amino acid metabolism and transport; F, nucleotide metabolism and transport; G, carbohydrate metabolism and transport; H, coenzyme metabolism, I, lipid metabolism; J, translation; K, transcription; L, replication and repair; M, cell wall/membrane/envelop biogenesis; N, cell motility; O, post-translational modification, protein turnover, chaperone functions; P, inorganic ion transport and metabolism; Q, secondary structure; T, signal transduction; U, intracellular trafficking and secretion; Y, nuclear structure; V, defense mechanisms; Z, cytoskeleton; W, extracellular structures; X, mobilome: prophages, transposons; R, general functional prediction only; S, function unknown. The color key reflects the total number of genes in each COG category.
